# ﻿Revision of *Troporhogas* Cameron (Hymenoptera, Braconidae, Rogadinae) with six new species from India and Thailand

**DOI:** 10.3897/zookeys.1206.120824

**Published:** 2024-07-05

**Authors:** Donald L. J. Quicke, A. P. Ranjith, Marisa K. Loncle, Cornelis Van Achterberg, Khuat Dang Long, Buntika A. Butcher

**Affiliations:** 1 Integrative Insect Ecology Research Unit, Department of Biology, Faculty of Science, Chulalongkorn University, Phayathai Road, Pathumwan, Bangkok 10330, Thailand Chulalongkorn University Bangkok Thailand; 2 Ashoka Trust for Research in Ecology and the Environment (ATREE), Royal Enclave, Srirampura, Jakkur Post, Bangalore 560064, India Ashoka Trust for Research in Ecology and the Environment (ATREE) Bangalore India; 3 Department of Terrestrial Zoology, Naturalis Biodiversity Center, Darwinweg 2, 2333 CR Leiden, Netherlands Naturalis Biodiversity Centre Leiden Netherlands; 4 Zhejiang University, Hangzhou 310058, China Zhejiang University Hangzhou China; 5 Institute of Ecology & Biological Resources (IEBR), Vietnam Academy of Science & Technology (VAST), 18 Hoang Quoc Viet Road, Cau Giay, Hanoi, Vietnam Institute of Ecology & Biological Resources (IEBR), Vietnam Academy of Science & Technology (VAST) Hanoi Vietnam

**Keywords:** Checklist, *Iporhogas*, ML phylogeny, new species, Rogadinae, Southeast Asia, *Troporhogas*

## Abstract

The genus *Troporhogas* Cameron, 1905 from the Indo-Malayan region is reviewed. Six new species, *Troporhogasalboniger* Quicke, Loncle & Butcher, **sp. nov.**, *T.benjamini* Quicke, Loncle & Butcher, **sp. nov**., *T.hugoolseni* Quicke, Loncle & Butcher, **sp. nov.**, *T.rafaelnadali* Quicke, Loncle & Butcher, **sp. nov.**, and *T.rogerfedereri* Quicke, Loncle & Butcher, **sp. nov.** from Thailand, and *T.anamikae* Ranjith, **sp. nov.** from India are described and illustrated photographically, bringing the total number of species of the genus known from the Indo-Malayan Region to 19. *Troporhogas* is recorded for the first time from India. A key is included to differentiate *Troporhogas* species. A four-gene ML tree based on COI, Cytb, 16S and 28S is reconstructed, representing the six new species. *Troporhogascontrastus* Long, 2014, originally described from Vietnam, is recorded from Thailand for the first time. The holotypes of the type species, *Troporhogastricolor* Cameron, 1905 and that of its junior synonym *Iporhogas* are illustrated, and photographs are presented of all the species known only from China and Sri Lanka. Sexual colour dimorphism of males of several species is described for the first time. Drawings summarising the different patterns of black marks on the metasoma that aid species recognition are presented.

## ﻿Introduction

The cosmopolitan Rogadinae Förster, 1863 is one of the most diverse subfamilies of Braconidae, with more than approximately 1,200 described species and 54 genera worldwide (Yu et al. 2016; Quicke et al. 2021). Of these, 16 Rogadinae genera occur in the New-World regions and 50 in the Old World tropics. Regarding its described species, it is the third most diverse subfamily in South East Asia (SEA) with 196 species known for Thailand (Songvorawit et al. 2021), while in contrast, only 36 species were reported for India (Yu et al. 2016; Ranjith et al. 2018, 2022; Rishabanu et al. 2021); the large former number is largely due to a single revisionary work on one huge genus by Butcher et al. (2012).

Rogadine wasps are koinobiont endoparasitoids that attack caterpillars in several groups (Zaldivar-Riverón et al. 2008; Quicke et al. 2021). When the rogadine wasp has completed its development, the host caterpillar is easily recognised because it is mummified and usually found attached to its host plant (van Achterberg 1991; Zaldivar-Riverón et al. 2008; Quicke 2015). This biological trait makes the subfamily particularly useful for the study of host-parasitoid associations, because the caterpillar’s remains can often be identified based on morphology (van Achterberg et al. 2020) or molecular techniques (Quicke et al. 2012). Unfortunately, there are no biological data as yet for the genus *Troporhogas*.

**Figure 1.**
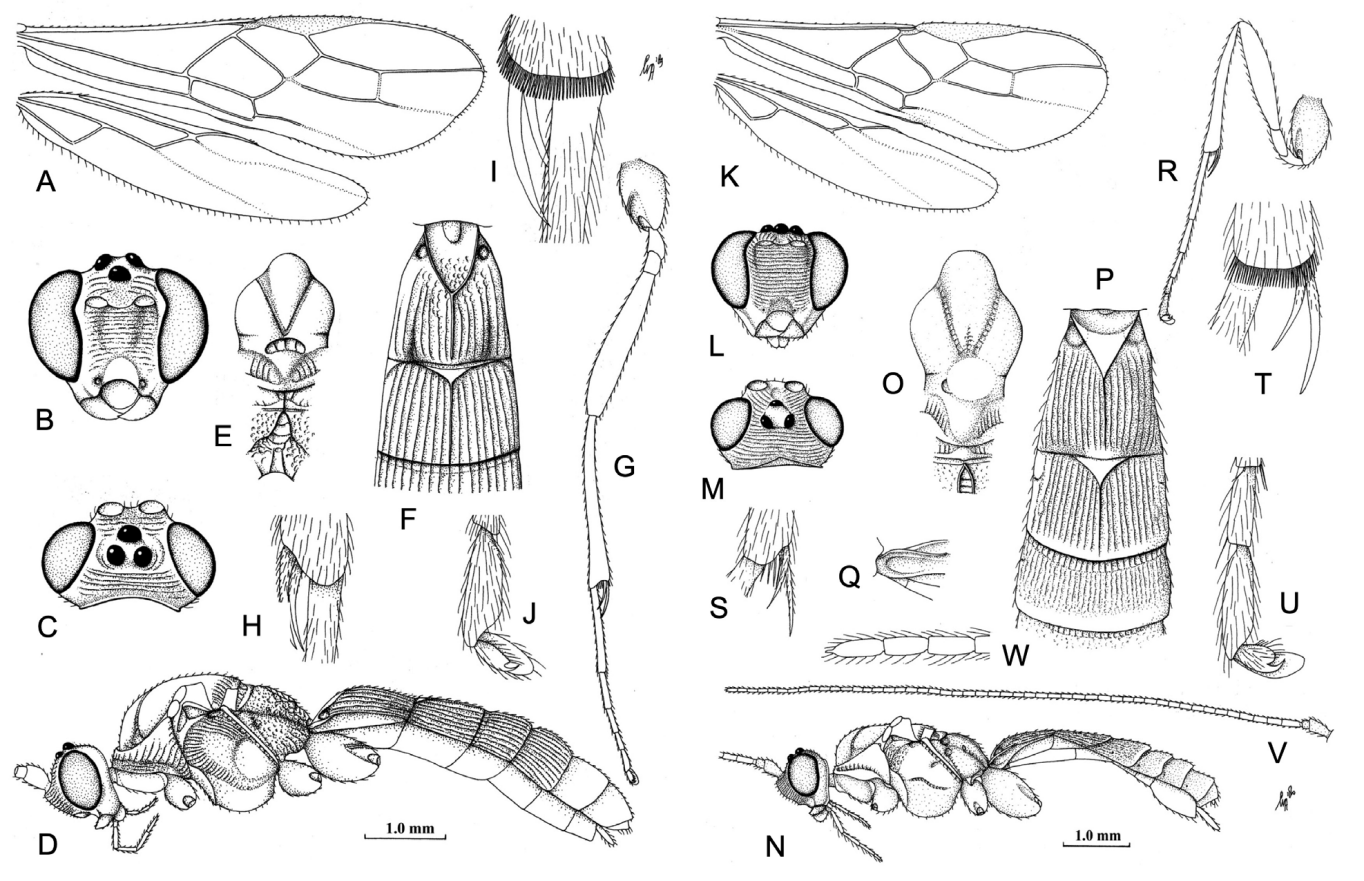
Drawings of lectotypes of **A–J***Troporhogastricolor* Cameron, the type species of the genus, and **K–W***T.infuscatipennis* (Granger), type species of *Iporhogas***A** wings **B** head, anterior view **C** head, dorsal view **D** habitus, lateral view **E** mesosoma, dorsal view **F** metasomal tergites 1 and 2, dorsal view **G** hind legs **H** middle tibial spurs **I** hind tibial spurs, inner aspect **J** outer hind claw **K** wings **L** head, anterior view **M** head, dorsal view **N** habitus, lateral view **O** mesosoma, dorsal view **P** metasomal tergites 1–3, dorsal view **Q** base of first tergite, lateral view **R** hind leg **S** middle tibial spurs, inner aspect **T** hind tibial spurs, inner aspect **U** outer hind claw **V** antenna **W** apical three flagellomeres.

Until recently, specimens from the Indo-Malayan region were referred to under the generic name *Iporhogas* Granger, 1949, which was originally described from Madagascar based on a single species. *Troporhogas* Cameron, 1905, was originally described from Sri Lanka; however, it was subsequently referred to only in catalogues, and species from SEA and southern China were described under the name *Iporhogas*. The two genera were formally synonymised by Quicke et al. (2021) based on a molecular phylogenetic study in which a Sri Lankan specimen of *T.ruficeps* Cameron, closely related to *T.tricolor*, the type species of *Troporhogas*, was deeply nested in a clade with species that had been assigned to *Iporhogas* (Chen and He 1997; Long 2014). We illustrate here the type species of the genera *Troporhogas* and *Iporhogas* here in Fig. 1. The type species of *Troporhogas*, *T.tricolor*, has a distinct mid-longitudinal carina on the median area of the metanotum (Fig. 1E), whereas this is absent in the type species of *Iporhogas* (Fig. 1O).

**Figure 2.**
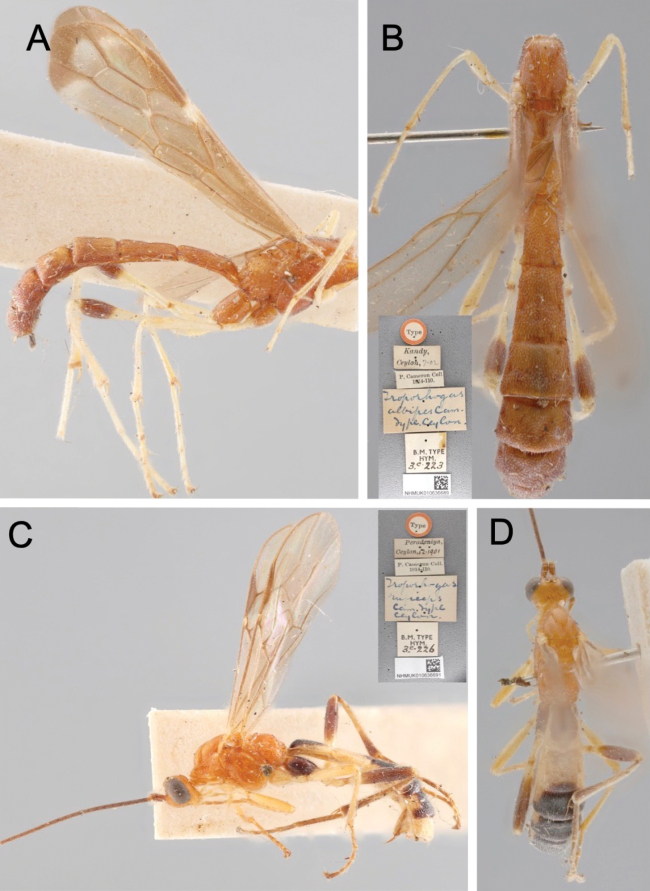
Light micrographs of ♀ holotypes of *Troporhogas* species described by Cameron (1905)**A***T.albipes* Cameron, lateral view **B***T.albipes*, dorsal view (labels inset) **C***T.ruficeps* Cameron, lateral view (labels inset) **D***T.ruficeps*, dorsal view.

**Figure 3.**
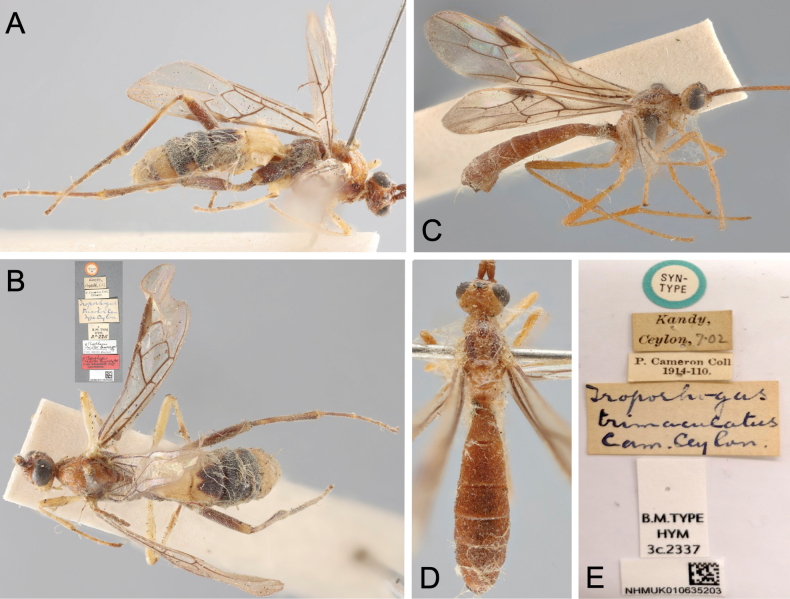
Light micrographs of *Troporhogas***A***T.tricolor* Cameron lectotype ♀, lateral view **B***T.tricolor* lectotype, dorsal view **C***T.trimaculatus* Cameron syntype ♀, lateral view **D***T.trimaculatus* syntype, dorsal view **E** labels of *T.trimaculatus* syntype.

*Troporhogas* was originally described based on seven species, all from Sri Lanka, viz.: *T.albipes*, *T.lateralis*, *T.maculipennis*, *T.ruficeps*, *T.spilonotus*, *T.tricolor*, and *T.trimaculatus* (Figs 2, 3). Of these, *T.maculipennis* was transferred to *Megarhogas* by Baltazar (1972). *Troporhogasspilonotus* was transferred to *Pseudogyroneuron* Baker, 1917, by Baltazar (1972) and then to *Canalirogas* van Achterberg & Chen, 1996, by Quicke and Shaw (2005) and subsequently treated as a senior synonym of *C.balgooyi* van Achterberg & Chen, 1996 (van Achterberg and Chen 1996) by Long and van Achterberg (2015). *Troporhogaslateralis* was formally transferred to *Rogas*, often regarded as a senior synonym of *Aleiodes*, and then to *Aleiodes* by Broad (2021); in the same paper, Broad designated a lectotype for *T.trimaculata*.

Members of *Troporhogas* can be recognised by the key provided in Chen and He (1997) in which they will key to *Iporhogas*. Many *Troporhogas* species have a distinctive habitus together with a bicoloured metasoma, black medially on some tergites (mainly on TT1–5) and white, especially on the anterolateral areas of the tergites (Long 2014), but in others it may be uniformly honey-coloured to ochraceous.

Chen and He (1997) recorded the genus for the first time from China, and described five new species, and Long (2014) recorded it from Vietnam and described an additional four species, all of these under the generic name *Iporhogas*.

Here we describe six more new species, *T.anamikae* sp. nov. from India, and *T.alboniger* sp. nov., *T.benjamini* sp. nov., *T.hugoolseni* sp. nov., *T.rafaelnadali* sp. nov., and *T.rogerfedereri* sp. nov. from Thailand, bringing the total number of *Troporhogas* species known worldwide to 20. This is the first report of *Troporhogas* from India and *T.contrastus* (Long 2014) and *T.tricoloratus* (Long 2014) are recorded from Thailand for the first time. A key is provided to enable recognition of all non-Afrotropical species. Further, since the original descriptions of the Chinese species, despite being quite thorough, were only illustrated with a few line drawings, we present photographs of the type specimens to facilitate use of the key. Phylogenetic relationships among all species of *Troporhogas* for which DNA data are available were also reconstructed based on four gene markers: cytochrome *c* oxidase subunit 1 (COI), cytochrome *b* (Cyt b), 16S rDNA, and the D2-D3 expansion region of 28S rDNA.

## ﻿Materials and methods

Specimens were collected with light traps at Doi Phu Kha National Park, Nan and Nakhon Ratchasima, with Malaise traps at Khao Yai National Park, Thailand, and by sweep net from secondary forests at Janakikkad, Kerala, south India.

The holotypes of *T.alboniger* sp. nov., *T.benjamini* sp. nov., *T.hugoolseni* sp. nov., *T.rafaelnadali* sp. nov. and *T.rogerfedereri* sp. nov. were imaged using a Leica M205 C with Montage multifocus, interactive measurement and fusion optics stereo microscope, using the Leica Application Suite (LAS). Holotype images of *T.anamikae* sp. nov. were taken using a Keyence VHX-6000 digital microscope. Raw figures were edited with the program GIMP v. 2.10.

### ﻿Repositories

**AIMB** ATREE Insect Collection, Ashoka Trust for Research in Ecology and the Environment, Bengaluru, Karnataka, India

**CUMZ**Insect Museum, Chulalongkorn University Museum of Natural History, Bangkok, Thailand

**NHMUK** Natural History Museum, London

**QSBG**Queen Sirikit Botanic Garden, Chiang Mai, Thailand

Terminology follows van Achterberg (1988) except for wing venation which follows Sharkey and Wharton (1997) and Butcher and Quicke (2023). Metasomal tergite/tergites are abbreviated as **T/TT**.

### ﻿Molecular methods

A molecular data matrix was created comprising up to four gene regions: barcoding region of cytochrome oxidase *c* subunit 1 (COI), cytochrome b (386 base pairs) (Cytb), regions IV and V of the mitochondrial 16S rRNA gene from H2507 to H1792′ (~ 650 bp), and the D2-D3 expansion region of 28S rDNA (28S). Most sequences (one sequence of the new species *T.benjamini*, *T.rafaelnadali*, and *T.rogerfedereri* and sequences of *T.contrastus*, *T.ruficeps*, *T.tricoloratus*, *Troporhogas* spp. 1–5, *Papuarogasdameni*, *Rogasodes* spp. 1 and 2, and *Rogasella* sp. 3) are taken from Quicke et al. (2021) and were generated from wasp legs by the Centre for Biodiversity Genomics, University of Guelph, based on standard protocols as described in Hebert et al. (2003), Park et al. (2010), and Quicke et al. (2023). In addition, we included sequences from representatives of three closely related species belonging to the genera *Papuarogas* Quicke, 2021, *Rhogasella* Baker, 1917, and *Rogasodes* Chen & He, 1997 as outgroups based on the large phylogeny presented by Quicke et al. (2021). Most of the Cytb sequences are newly generated.

Alignment of both COI and cytochrome b sequences was trivial as there were no indels. The length-variable 28S sequences were aligned according to the secondary structure model of Gillespie et al. (2005) as in other studies (Butcher et al. 2014; Quicke et al. 2016) and the length-variable 16S sequences were aligned according to the secondary structure models (Buckley et al. 2000; Wu et al. 2014). For both COI and Cytb, the three codon positions were each treated as a separate partition. For both 16S and 28S, only confidently aligned base pairs were included and each was treated as a single partition.

Maximum likelihood (ML) trees were constructed using RAxML v. 7.0.4 (Stamatakis 2014) with the GTRGAMMA model and a rapid bootstrap (100 replicates) using the options -m GTRGAMMA -f a -# 100 command. Tree was visualised using FigTree v. 1.4.4. (Rambaut 2018).

Provenances of sequenced specimens, DNA barcode index numbers (BINs), and GenBank accessions numbers are given in Suppl. material 1. In addition, the colour pattern on metasomal tergite of *Troporhogas* is provided in Fig. 21.

## ﻿Results

### ﻿Checklist and distribution of *Troporhogas* species worldwide

*Troporhogasanamikae* Ranjith, sp. nov., India

*Troporhogasalbilateralis* (Long, 2014) (= *Iporhogasalbilateralis* Long, 2014), Vietnam, Thailand)

*Troporhogasalbipes* Cameron, 1905, Sri Lanka

*Troporhogasalboniger* Quicke, Loncle & Butcher, sp. nov., Thailand

*Troporhogasbenjamini* Quicke, Loncle & Butcher, sp. nov., Thailand

*Troporhogaschinensis* (Chen & He, 1997), (= *Iporhogaschinensis* Chen & He, 1997), China

*Troporhogascontrastus* (Long, 2014), (= *Iporhogascontrastus* Long, 2014), Vietnam, Thailand

*Troporhogasflavistigma* (Chen & He, 1997), (= *Iporhogasflavistigma* Chen & He, 1997), China

*Troporhogasguangxiensis* (Chen & He, 1997), (= *Iporhogasguangxiensis* Chen & He, 1997), China, Vietnam

*Troporhogashugoolseni* Quicke, Loncle & Butcher, sp. nov., Thailand

*Troporhogasinfuscatipennis* (Granger, 1949), (= *Iporhogasinfuscatipennis* Granger, 1949), Madagascar

*Troporhogasrafaelnadali* Quicke, Loncle & Butcher, sp. nov., Thailand

*Troporhogasrogerfedereri* Quicke, Loncle & Butcher, sp. nov., Thailand

*Troporhogasruficeps* Cameron, 1905, Sri Lanka

*Troporhogasrugivertex* (Chen & He, 1997), (= *Iporhogasrugivertex* Chen & He, 1997), China

*Troporhogassimulatus* (Long, 2014), (= *Iporhogassimulatus* Long, 2014), Vietnam

*Troporhogastricolor* Cameron, 1905, Sri Lanka

*Troporhogastricoloratus* (Long, 2014), (= *Iporhogastricoloratus* Long, 2014), Vietnam

*Troporhogastrimaculatus* Cameron, 1905, Sri Lanka

*Troporhogasunicolor* (Chen & He, 1997), (= *Iporhogasunicolor* Chen & He, 1997), China, Thailand

### ﻿Phylogeny

**Figure 4.**
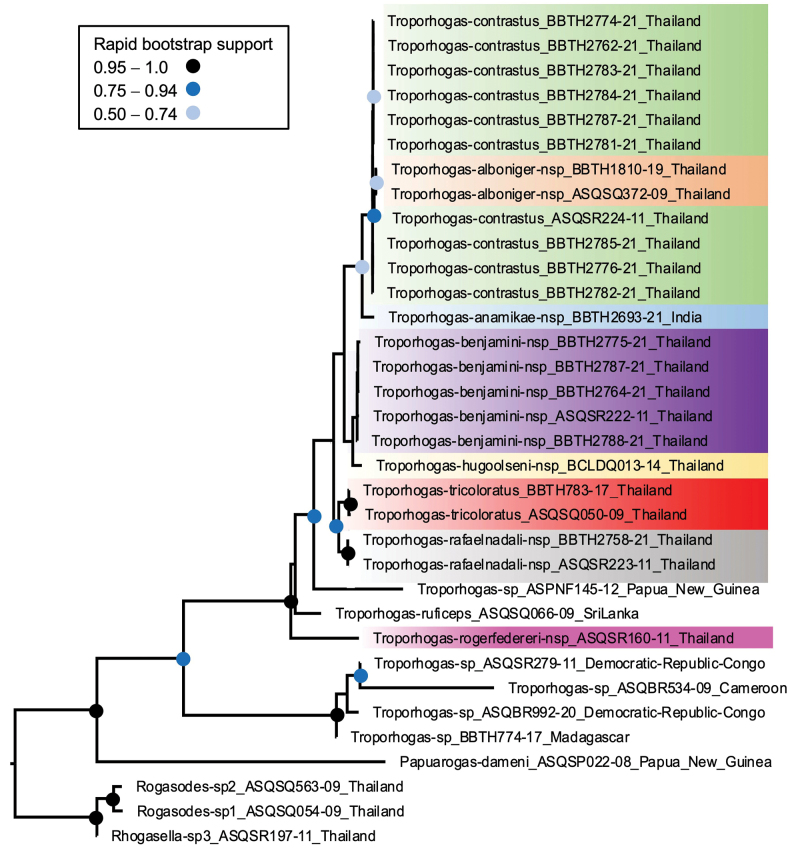
Maximum likelihood tree based on combined analysis of four gene fragments (COI, Cytb, 16S, 28S) using GTR+G parameter model with *Troporhogas* represented by 31 BINS. Support values at nodes are rapid bootstrap and indicated by coloured dots.

The maximum likelihood tree obtained is shown in Fig. 4. *Troporhogas* was recovered as monophyletic with 100% bootstrap support. The circles at nodes indicate rapid bootstrap clade support. The genus comprised two separate clades each with 100% bootstrap support, one including all the Afrotropical species and the other all Indo-Australian species. Six of the Thai species and the one from India form a monophyletic group with 79% bootstrap support. Of these, *T.rafaelnadali* sp. nov. and *T.tricoloratus* were both supported ≥ 99%. The most basal member of the Asian clade was *T.rogerfedereri* sp. nov., a species that appears most closely related, on the basis of morphology, to the Chinese *T.flavistigma*, both sharing five carinae in the scutellar sulcus and anteriorly widely-separated submedial longitudinal propodeal carinae anteriorly (Figs 16G, 19F). However, within the remainder of this clade there was little support for the additional species recognised by morphological characters, especially for *T.alboniger* sp. nov. and *T.contrastus* Long and our reasons for recognising this new species are dealt with below.

### ﻿Systematics

#### ﻿Class Insecta Linnaeus, 1758

**Order Hymenoptera Linnaeus, 1758**

**Family Braconidae Nees, 1811**

**Subfamily Rogadinae Foerster, 1863**

##### ﻿ Troporhogas

Taxon classificationAnimaliaHymenopteraBraconidae

Cameron, 1905

A6F7DB61-3908-558A-8F17-D3277A8858CF

Troporhogas Cameron, 1905: 92 (type species Troporhogastricolor Cameron, 1905).
Iporhogas Granger 1949: 167; synonymised by Quicke et al. 2021: 16.

###### Generic diagnosis.

Antenna usually ~ 1.5 × fore wing length; palpi normal; face at least with some transverse striation; malar suture shallow; eyes emarginate; temple with fine striation; frons rather flat, usually with transverse of oblique striation, usually with a pair of carinae running posteriorly from the lateral margins of antennal sockets and then converging more or less joining anterior to median ocellus separating frons into anterior and posterior portions (Figs 15B, C, 17B); occipital carina complete, joining hypostomal carina ventrally well-removed from base of mandible; mesosoma largely smooth shiny; notauli moderately deep and crenulate, converging but not uniting posteriorly, not reaching posterior margin of mesoscutum; prepectal carina complete or nearly so; precoxal sulcus present; scutellar sulcus wide with single mid-longitudinal carina; metanotum with mid-longitudinal carina at least posteriorly, though sometimes indistinct; propodeum areolate, with at least a trace of submedial carinae close together anteriorly, gradually diverging posteriorly; tarsal claws usually with small to large, pointed or angulate basal lobe (except absent in *T.guangxiensis* and *T.simulatus*); fore wing vein 1m-cu antefurcal, slightly curved, gradually merging into vein 1CUb; 2^nd^ submarginal cell elongate, vein 2RS > 1.7 × width of cell; hind wing vein 1M straight, approximately as long as M+CU; hind wing vein RS weakly curved basally and only short basal stub, tubular and sclerotised; hind wing vein m-cu absent; hind wing veins M+CU and 1M of approximately same length; middle tibial spurs largely setose and nearly straight; apex of hind tibia with distinct comb of specialised setae medially; hind tibial spurs curved and at least apical half glabrous; T1 not widened basally, with large dorsope, dorsal carinae united behind level of spiracles to form complete mid-longitudinal carina, and without pair of submedial carinae; T2 with distinct mid-basal triangular area, giving rise to complete or nearly complete mid-longitudinal carina; TT3–6 without mid-longitudinal carina; TT2–5 with sharp lateral crease, largely finely longitudinally striate; hypopygium large, ventrally slightly convex and apically truncate; ovipositor sheath rather slender.

###### Type species.

*Troporhogastricolor* Cameron, 1905 (Fig. 3).

###### Diagnosis.

Antenna longer than body, slender, setose, 40–50 flagellomeres. Eyes large, clearly emarginated on inner side; malar space short (Fig. 3B). Maxillary palp very long, slender, setose, 4–5 jointed (Fig. 3D). Temple short, oblique. Occiput sharply margined, transverse (Fig. 3C). Metanotum with two roundly diverging carina basally (Fig. 3E). Mesopleuron with a depression ventrally (Fig. 3D). Legs long and slender; femora narrowed basally (Fig. 3J). Fore wing 2^nd^ submarginal cell 2.0 × longer than wide, of equal width throughout; anal vein not interstitial (Fig. 3A); vein (RS+M)b short; vein r-rs less half the length of 3RSa; vein 3RSb longest and curved upwards (Fig. 3A). Tarsi longer than tibiae; basitarsus longer than two following joints combined (Fig. 3J). Hind tibial spurs glabrous (Fig. 1I). Metasoma 2.0 × as long as mesosoma (Fig. 3D); TT1–3 closely longitudinally striated (Fig. 3G); TT4–6 with posterior transverse furrows; base of TT4–6 depressed, apex of TT4–6 raised and clearly separated from the base. Hypopygium large, cultriform; ovipositor shortly projecting, the sheaths stout (Fig. 3D). Head rufous; mesosoma largely orange with propodeum largely piceous; metasoma cream-white with medial black mark on T2, with larger black marks on TT3 and 4 which reach lateral margins posteriorly.

### ﻿Key to the species of Indo-Malayan *Troporhogas*

**Table:** 

1	Tarsal claws simple, without lobe; hind tibial spurs entirely glabrous	**2**
–	Tarsal claws with minute to large lobe; hind tibial spurs setose basally	**3**
2(1)	Occipital carina in dorsal view angularly concave; stemmaticum without any dark mark; hind wing vein SC+Ra almost straight apically; vein SC+Rb of hind wing quadrate; TT1–3 ochreous yellow, 4–6 paler yellow. Vietnam	***T.simulatus* Long**
–	Occipital carina in dorsal view roundly concave; propodeum without areola and with basal carina; hind wing vein SC+Ra distinctly curved apically; hind wing vein SC+Rb subquadrate, swollen apically; metasomal tergites yellowish with pale brown patches anteromedially (Fig. 19H). China, Vietnam	***T.guangxiensis* Chen & He**
3(1)	Metasoma honey to brownish yellow with or without darker marks or paler marks (Figs 2A, B, 3D, 19B, D, H)	**4**
–	Metasoma white or pale cream with black marks (Fig. 20)	**7**
4(3)	Wing membrane patterned hyaline and grey (Figs 2A, 3C)	**5**
–	Wing membrane plain and uniform (e.g. Figs 5D, 9B)	**6**
5(4)	Hind femur robust (Fig. 2A); hind leg boldly patterned, white with distal 0.7 femur dark brown (Fig. 2A); mesoscutum uniformly brown-yellow (Fig. 2B). Sri Lanka	***T.albipes* Cameron**
–	Hind femur slender (Fig. 3C); hind leg uniformly brown-yellow (Fig. 3C); mesoscutum with three dark marks (Fig. 3D). Sri Lanka	***T.trimaculatus* Cameron**
6(4)	Occipital carina in dorsal view strongly angularly (Fig. 19C); vertex smooth; fore wing veins all pale yellow. China	***T.unicolor* Chen & He**
–	Occipital carina in dorsal view weekly rounded; vertex transversely rugose; fore wing veins 1M, 1CUb and 1cu-a darker brown than remaining venation. China	***T.rugivertex* Chen & He**
7(3)	Submedial propodeal carinae anteriorly forming a wide inverted ‘U’-shape (Figs 16G, 19F); scutellar sulcus with five carinae between outer borders (Figs 16G, 19F); T5 variable, often largely or entirely cream-white (Fig. 18D, F); vertex largely smooth except immediately posterior to stemmaticum	**8**
–	Submedial propodeal carinae anteriorly running closer together and forming an inverted narrow or wider V-shape (Fig. 14A); scutellar sulcus usually with three carinae between outer borders (Figs 14A, 15F, 19B); T5 largely black except for anterolateral areas (Figs 2C, D, 15G, 16D, 17H, 18C); vertex variable	**9**
8(7)	Occipital carina medially forming a point (Fig. 19G); vertex largely smooth except immediately posterior to stemmaticum; pterostigma largely yellow-brown and venation pale (Fig. 19G); dark mark on T1 not reaching posterior margin (Fig. 19H). China	***T.flavistigma* Chen & He**
–	Occipital carina medially more rounded (Fig. 16C); vertex (extending well towards temples) strongly transversely striate (Fig. 16D); pterostigma black (Fig. 16I); dark mark on T1 reaching posterior margin (Fig. 19H). Thailand	***T.rogerfedereri* sp. nov.**
9(7)	T1 entirely white (Figs 3A, B, 6B, 9D)	**10**
–	T1 with a well-developed black mark at least anteriorly (Figs 2C, D, 13A, 16D)	**14**
10(9)	Head, mesosoma entirely black except for white tegulae (Figs 5A, 6A); T2 entirely white (Fig. 6B). Thailand	***T.alboniger* sp. nov.**
–	Head, mesoscutum and pronotum with yellow marks, at least at mid-posterior of mesoscutum and scutellar sulcus (Figs 12C) or mesosoma largely pale (Fig. 3B)	**11**
11(10)	T4 medially and T5 entirely white (Fig. 9A, B). India	***T.anamikae* sp. nov.**
–	TT4 and 5 almost entirely black except for white anterolateral areas	**12**
12(11)	T5 largely whitish (Fig. 3A, B). Sri Lanka	***T.tricolor* Cameron**
–	T5 black (Figs 12B, 17B)	**13**
13(12)	Vertex and temples finely transversely striate (Fig. 11C); mesoscutum mainly black except medial-posteriorly white (Fig. 12A). Thailand	***T.benjamini* sp. nov.**
–	Vertex and temples polished, without conspicuous striation; mesoscutum laterally black, middle lobe entirely brownish yellow (occasionally piceous on anterior half). Thailand, Vietnam	***T.contrastus* Long**
14(9)	Dark markings on TT1–5 contiguous (Figs 19D, 21D); vertex finely transversely sculptured. China	***T.chinensis* Chen & He**
–	Dark markings on TT1–5 not contiguous; vertex sculpture variable	**15**
15(14)	Vertex with setiferous punctation but without distinct transverse striation (Fig. 13C). Thailand	***T.hugoolseni* sp. nov.**
–	Vertex transversely striate (Fig. 18D)	**16**
17(16)	Raised oblique area of mesopleuron immediately below subalar depression strongly finely striate (Fig. 15E). Thailand	***T.rafaelnadali* sp. nov.**
–	Raised oblique area of mesopleuron immediately below subalar depression smooth, without striation. Thailand, Vietnam	***T.tricoloratus* Long**

### ﻿Descriptions

#### ﻿ Troporhogas alboniger

Taxon classificationAnimaliaHymenopteraBraconidae

Quicke, Loncle & Butcher sp. nov.

61D3EAFB-85BB-5905-8121-4213603AA629

https://zoobank.org/1ED0F077-8612-4593-878F-A39CEEFCD34A

**Figure 5.**
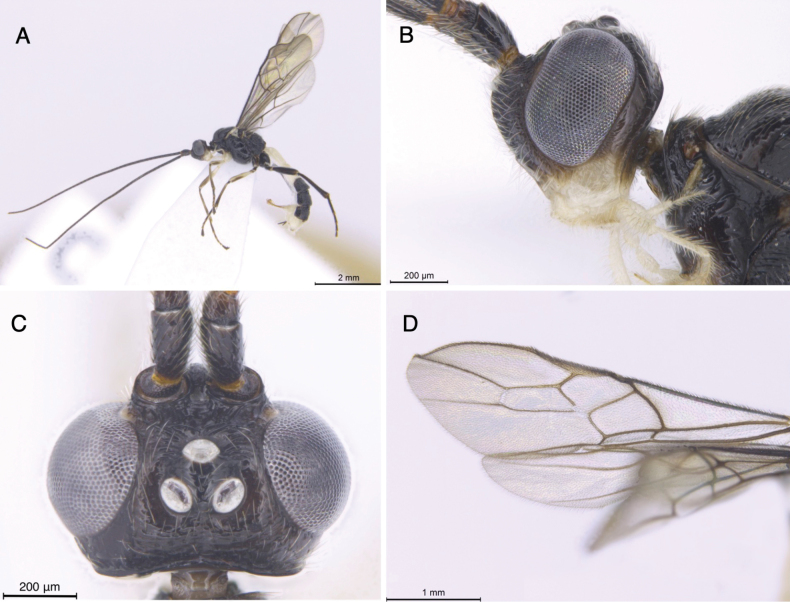
Light micrographs of holotype female *T.alboniger* sp. nov. **A** habitus, lateral view **B** head, lateral view **C** head, dorsal view **D** fore wing.

**Figure 6.**
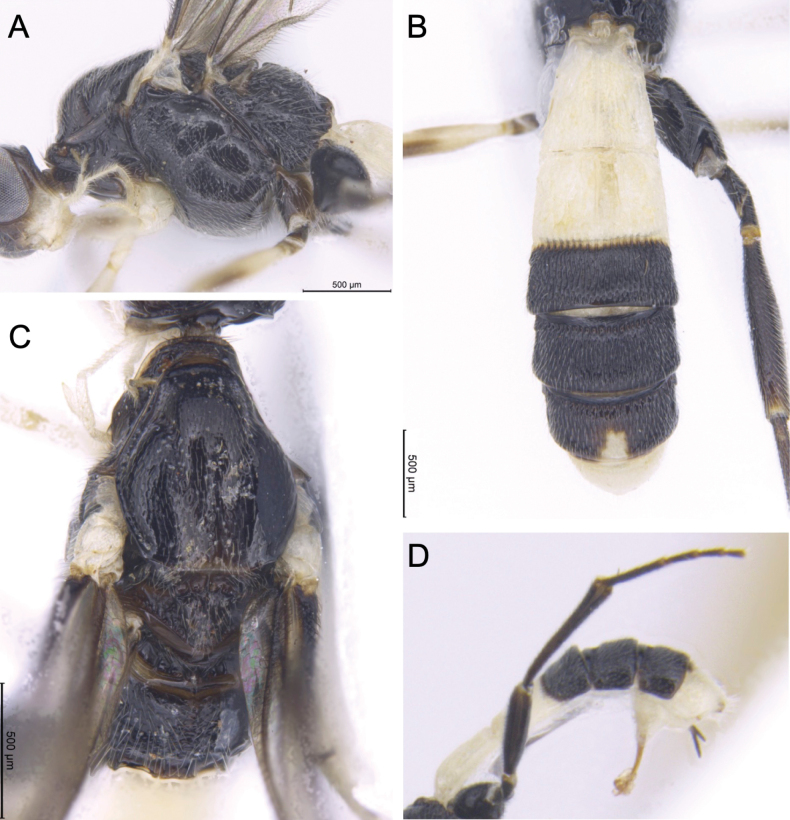
Light micrographs of holotype female *T.alboniger* sp. nov. **A** mesosoma, lateral view **B** metasoma, dorsal view **C** mesosoma, dorsal view **D** metasoma, lateral view.

**Figure 7.**
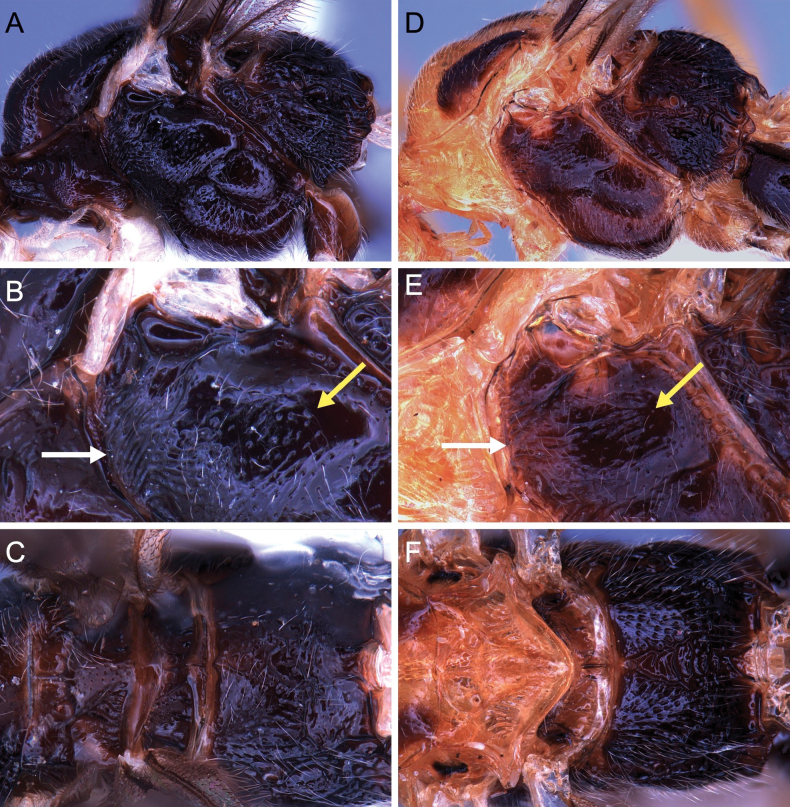
Light micrographs of holotype female *T.alboniger* sp. nov. comparing with Thai specimen of *T.contrastus***A** mesosoma of *T.alboniger* sp. nov., lateral view **B** mesopleuron of *T.alboniger* sp. nov. **C** scutellum and propodeum of *T.alboniger* sp. nov., dorsal view **D** mesosoma of *T.contrastus*, lateral view **E** mesopleuron of *T.contrastus***F** scutellum and propodeum of *T.contrastus*, dorsal view.

Figs 5 , 6 , 7A–C , 21J

##### Type material.

***Holotype*** ♀. Thailand, Nan province, Doi Phu Kha National Park, 19°12.164'N, 101°04.473'E, 19.vi.2019, M.V. light trap, col. M. Loncle (CUMZ). ***Paratype*** Thailand: ♂ Loei province, Phu Ruea National Park, 17°28.826'N, 101°21.330'E, 19–26.vii.2006, Malaise trap, col. N. Jareonchai (QSBG).

##### Diagnosis.

*Troporhogasalboniger* sp. nov. can be differentiated from all other species by its colour pattern, the female being bicoloured (black and white), its body without any yellow or brownish yellow colouration. Head black except area around the mouthparts including palps white. The mesosoma is completely black except for white tegular area. Metasoma bicoloured: TT1, 2, and 6 white, TT3–5 black (with white spot on the middle of posterior area of fifth tergite). Molecularly (Fig. 1) and morphologically it is closest to *T.contrastus* (originally described from Vietnam) which displays a very different colour pattern, having the head and anterior mesosoma yellow. Apart from colouration, *T.alboniger* sp. nov. differs from *T.contrastus* in (1) having fewer flagellomeres (41 cf. 45, 46); (2) anterior margin of mesopleuron a little below antescutal depression with zone of fine subvertical striation with (Fig. 7B, E; white arrows) compared with a few, irregular somewhat longitudinal rugae (Fig. 7B, E; white arrows); (3) posteromedial part of mesopleuron above precoxal sulcus with more or less round setiferous pits (Fig. 7B, E; yellow arrows) compared with distinctly elongate pits (Fig. 7B, E; yellow arrows); (4) anteromedial area of propodeum adjacent to anterior areola with smaller and more widely spaced setiferous punctures (Fig. 7C) compared with a denser zone of deep large setiferous punctures (Fig. 7F).

##### Description.

Holotype, female, body length 5.5 mm; fore wing 4.6 mm; ovipositor sheath 0.6 mm.

***Head.*** Antenna with 41 flagellomeres. Terminal flagellomere acuminate, 4 × longer than wide, 1.3 × longer than penultimate flagellomere. First flagellomere 1.1 × and 1.25 × longer than second and third, respectively, the latter 2.1 × longer than wide. Width of head: width of face: height of eye = 2.3: 1.0: 1.1. Face and clypeus rugose with sparse setosity laterally. Inter-tentorial distance 2.2 × tentorio-ocular distance. Shortest distance between posterior ocelli: transverse diameter of posterior ocellus: shortest distance between posterior ocellus and eye = 1.0: 2.3: 2.0. Vertex and temple smooth and shiny, with distinct mid-longitudinal groove. Occipital carina very weakly curved dorsally, a distinct transverse groove present immediately anterior to it.

***Mesosoma.*** Mesosoma 1.6 × longer than high. Notauli crenulate becoming foveate posteriorly. Scutellar sulcus with three well-developed carinae between outer pair. Axilla with ~ 6 sinuous carinae carina. Mesopleuron and metapleuron setose, precoxal sulcus a short deep groove with a broad area of irregular striation extending below it. Propodeum with submedial carinae weak, uniting anteriorly in a narrow U-shape, lateral to this with long setae, arising from distinct small punctures.

***Wings.*** Fore wing. Length ratios of fore wing veins r-rs: 3RSa: 3RSb = 1.0: 3.2: 5.1. Length ratios of vein 2RS: 3RSa: rs-m = 2.2: 2.9: 1.0.

***Legs.*** Length ratios of fore femur: fore tibia: fore tarsus = 1.1: 1.0: 1.1. Length ratios of hind femur: hind tibia: hind tarsus = 1.0: 1.0: 1.0. Length of hind femur and tibia 4.5 × and 8.6 × as long as medially wide, respectively. Tarsal claws with large acutely pointed basal lobe.

***Metasoma.*** T1 1.2 × longer than posteriorly wide. T2 1.4 × longer than third. TT1 and 2 with mid-longitudinal carina dorsally, sparsely striate. Ovipositor sheath straight and shorter than hind basitarsus, ~ 0.3 × length hind femur (including trochantellus).

***Colour.*** Bicoloured body; scapus, pedicellus and antennal segments black. Head brownish black, but mouthpart area and palps white, stemmaticum black. Mesosoma dark brown to black except for white tegulae. Metasoma bicoloured; TT1,2, and 6 white, TT3–5 black with white spot on the middle of posterior area of T5, hypopygium white. Wings hyaline with brown venation, pterostigma brown becoming pater posteriorly. Fore and mid legs pale brown, paler posteriorly, and coxa and trochantellus white; hind legs entirely black. Ovipositor sheath black.

**Male.** Length of body 4.6 mm, of fore wing 4.1 mm. Antenna incomplete, with at least 34 flagellomeres. Occipital carina distinctly pointed medially. Head and mesosoma yellow. Metasomal tergites white except TT3 and 4 which each have a pair of black submedial, somewhat triangular marks.

##### Distribution.

North and east Thailand.

##### Biology.

Unknown.

##### Etymology.

From Latin *alboniger* referring to the white and black colouration.

#### ﻿ Troporhogas anamikae

Taxon classificationAnimaliaHymenopteraBraconidae

Ranjith sp. nov.

E4E9CC0B-5071-5F07-BF8C-891C480A5B6D

https://zoobank.org/79017778-9048-4E70-92CE-371F07A6176C

**Figure 8.**
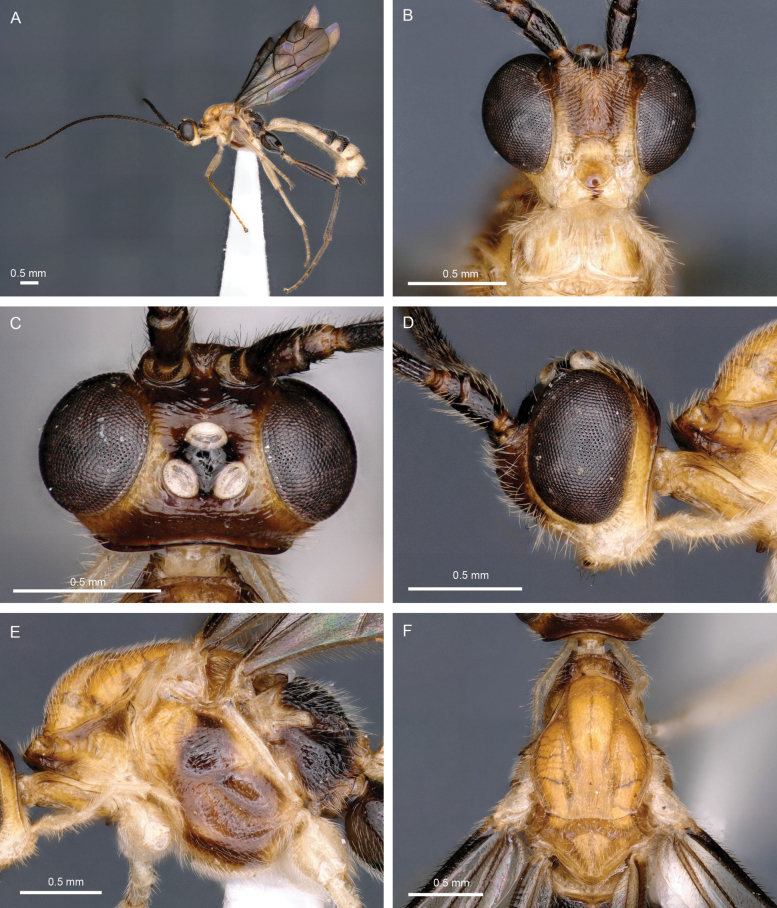
Light micrographs of holotype female *T.anamikae* sp. nov. **A** habitus, lateral view **B** head, anterior view **C** head, dorsal view **D** head, lateral view **E** mesosoma, lateral view **F** propodeum dorsal view.

**Figure 9.**
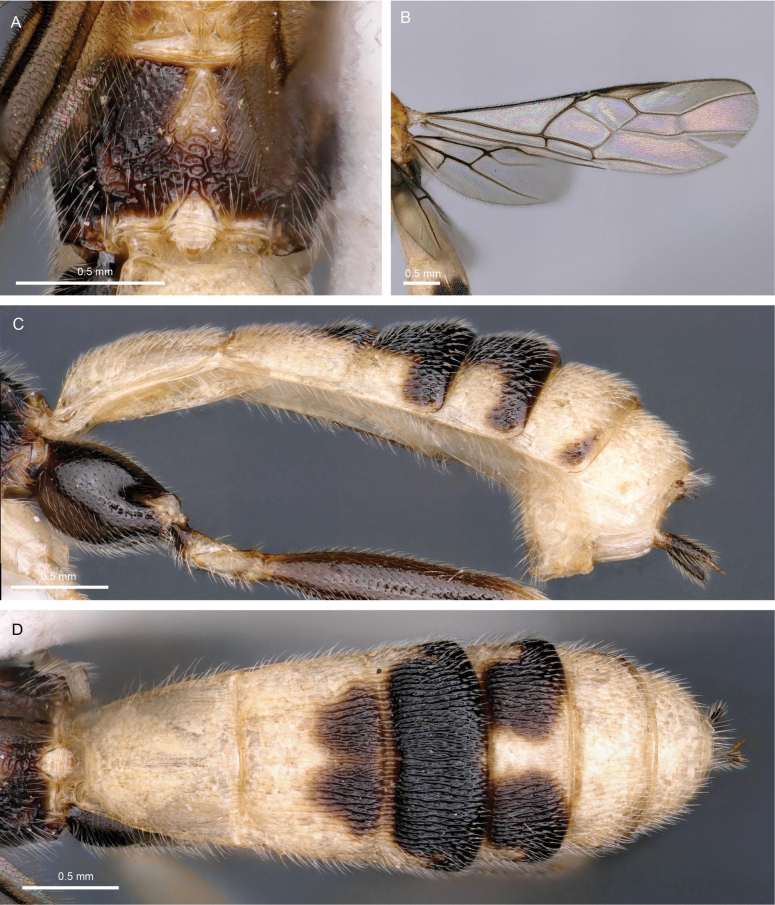
Light micrographs of holotype female *T.anamikae* sp. nov. **A** propodeum, dorsal view **B** wings **C** metasoma, lateral view **D** metasoma, dorsal view.

**Figure 10.**
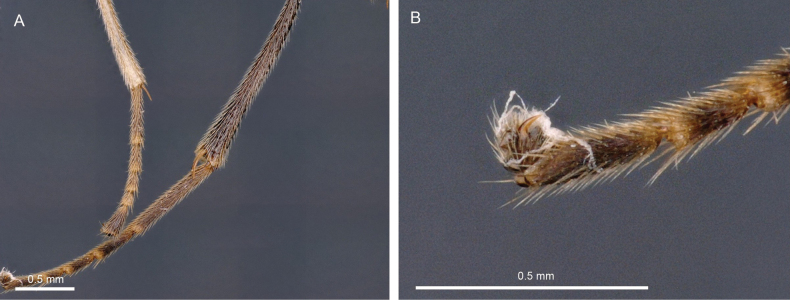
Light micrographs of holotype female *T.anamikae* sp. nov. **A** hind tibia **B** hind tarsus.

Figs 8 , 9 , 10 , 21K

##### Type material.

***Holotype*** ♀, India • Kerala, Kozhikode, Janakikkad, 3.i.2020, 11°37.309'N, 75°47.308'E, sweep net, col. Ranjith, A.P. (AIMB).

##### Diagnosis.

*Troporhogasanamikae* sp. nov. is similar to *T.tricolor* Cameron in having a more or less similar colour pattern but the new species differs in having notauli distinct only in the basal half of mesoscutum (vs notauli complete in *T.tricolor*), mesosoma yellow dorsally (vs black in *T.tricolor*), pterostigma mostly yellow (vs completely black in *T.tricolor*), face with smooth longitudinal area antero-medially (vs completely striated in *T.tricolor*), ocello-ocular area smooth (vs striated in *T.tricolor*), midbasal triangular area of propodeum smooth (vs irregularly reticulated in *T.tricolor*), T5 longitudinally striated (vs punctate in *T.tricolor*).

##### Description.

Holotype, female, body length 6.2 mm, fore wing 5.1 mm, ovipositor sheath 0.4 mm.

***Head.*** Antenna with 45 flagellomeres. First flagellomere 1.6 × longer than second and third, respectively. Width of head: width of face: height of eye = 2.75: 1.0: 1.5. Shortest distance between posterior ocelli: transverse diameter of posterior ocellus: shortest distance between posterior ocellus and eye = 0.7: 2.0: 1.0. Face transversely striate with a smooth longitudinal area antero-medially. Clypeus smooth with sparse setosity laterally. Vertex and temple finely transversely striate. Occipital carina complete, very weakly curved dorsally.

***Mesosoma.*** Mesosoma 1.5 × longer than high, more or less smooth to coriaceous and setose. Mesoscutum smooth without groove medially, notauli present, crenulated. Scutellar sulcus smooth with seven carinae. Mesopleuron and metapleuron setose, precoxal sulcus broad, upwardly curving, and striate. Propodeum reticulate with long setae and without a mid-longitudinal carina.

***Wings.*** Fore wing. Lengths of fore wing veins r-rs: 3RSa: 3RSb = 1.0: 2.5: 5.0. Lengths of vein 2RS: 3RSa: rs-m = 1.3: 2.4: 1.0.

***Legs.*** Lengths of fore femur: fore tibia: fore tarsus = 1.0: 1.1: 1.1. Lengths of hind femur: hind tibia: hind tarsus = 1.0: 1.2: 1.5. Length of hind femur and tibia 5.0 × and 8.5 × as long as wide, respectively. Tarsal claws with large acutely pointed basal lobe.

***Metasoma.*** T1 as long as posteriorly wide. T2 1.6 × longer than third tergite. TT1 and 2 with medial longitudinal carina dorsally, distinctly striate. TT3 and 4 distinctly striate, TT5 and 6 finely longitudinally striate. Ovipositor sheath straight and shorter than hind basitarsus, ~ 0.2 × length of hind femur (including trochantellus).

***Colour.*** Body mostly yellow except antenna, eye, face medially, tip of mandible, frons, ocellar region, occiput, mesopleuron posterior 0.7, metapleuron, propodeum, wing venation, hind leg except trochanter, T2 with bilobed black mark mid-posteriorly, T3 largely except anterolateral areas, T4 with pair of large black marks submedially which reach posterior and posterolateral margin of tergite. Ovipositor sheath brown.

**Male.** Unknown.

##### Distribution.

Kerala (India).

##### Host.

Unknown.

##### Etymology.

APR dedicates this species to his friend Ms Anamika Menon, for her constant support and encouragement.

#### ﻿ Troporhogas benjamini

Taxon classificationAnimaliaHymenopteraBraconidae

Quicke, Loncle & Butcher sp. nov.

59C2CFAE-870D-5D4B-974C-8742F670017E

https://zoobank.org/47B75022-5A59-4CE5-9333-C72AE3E58B9A

Figs 11 , 12 , 21H

##### Material.

***Holotype*** ♀, Thailand, Nakhon Ratchasima province, Wang Nam Khaew district, Sakaerat Environmental Research Station (SERS), 26.vii.2021, 14°60.755'N, 101°82.761'E, M.V. light trap, col. K. Chansri (CUMZ). ***Paratypes*** Thailand: 1♂, Nakhon Ratchasima province, Wang Nam Khaew district, Sakaerat Environmental Research Station (SERS), 26.vii.2021, 14°60.755'N, 101°82.761'E, M.V. light trap, col. K. Chansri (CUMZ); 1♀, Nakhon Ratchasima province, Wang Nam Khaew district, Sakaerat Environmental Research Station (SERS), 25.i.2021, 14°49.672'N, 101°91.615'E, aerial net, col. K. Chansri (CUMZ); 1♀, Nakhon Ratchasima province, Wang Nam Khaew district, Sakaerat Environmental Research Station (SERS), 30.viii.2021, 14°49.630'N, 101°91.600'E, Malaise trap, col. K. Chansri (CUMZ); 1♀, Phetchaburi province, Kaeng Krachan National Park, 3–10.iv.2009, 12°48.107'N, 99°26.669'E, Malaise trap, col. Sirichai (CUMZ).

**Figure 11.**
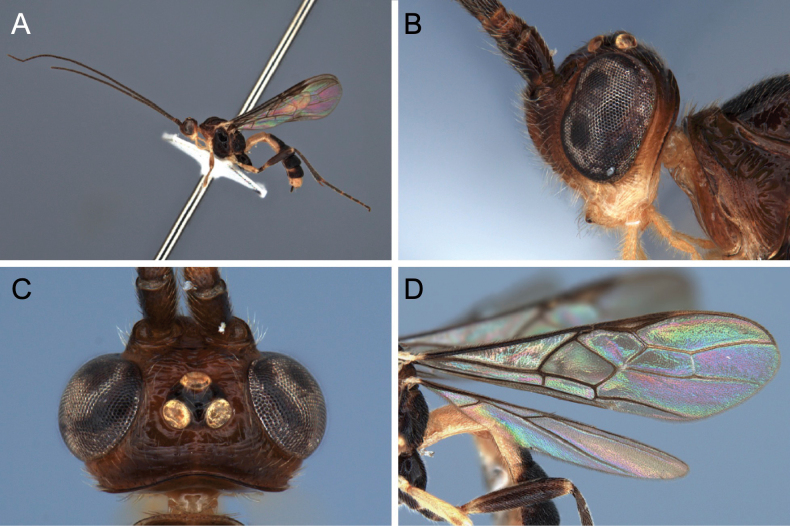
Light micrographs of holotype female *T.benjamini* sp. nov. **A** habitus, lateral view **B** head, lateral view **C** head, dorsal view **D** fore wing.

##### Diagnosis.

**Figure 12.**
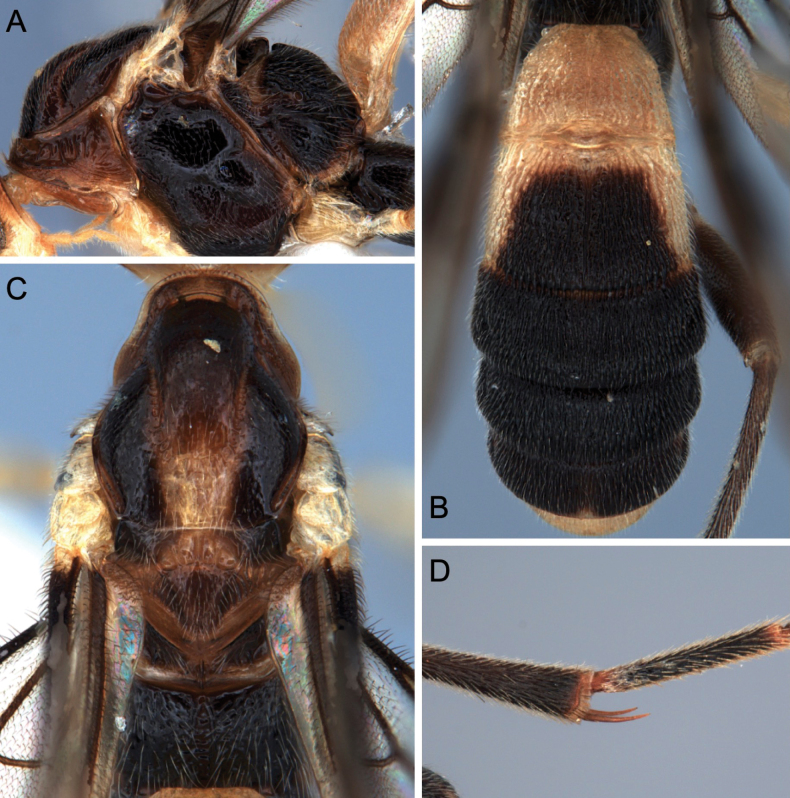
Light micrographs of holotype female *T.benjamini* sp. nov. **A** mesosoma, lateral view **B** metasoma, dorsal view **C** mesosoma, dorsal view **D** hind tibial spurs.

*Troporhogasbenjamini* sp. nov. can be differentiated from others species by its colour pattern, which is mainly dark brownish red with ivory white on mouthparts including palps, tegula (Fig. 12C) and TT1 and 2 (anteriorly and laterally) and T6. Metasoma bicoloured: T1 and T6 ivory, T2 anteriorly and laterally ivory with black mark in the middle, TT3–5 mainly black except for small anterolateral areas.

##### Description.

Holotype, female body length 5.5 mm; fore wing 4.9 mm; ovipositor sheath 0.05 mm.

***Head.*** Antenna with 50 flagellomeres. Terminal flagellomere acuminate, 3.7 × longer than wide, 1.4 × longer than penultimate flagellomere. First flagellomere 1.2 × longer than second and third, respectively. Width of head: width of face: height of eye = 3.2: 1.5: 1.0. Inter-tentorial distance 3.2 × tentorio-ocular distance. Face and clypeus rugose with sparse setosity laterally. Frons with strong transverse striation that obscure the transverse carina arising from outer part of antennal sockets. Shortest distance between posterior ocelli: transverse diameter of posterior ocellus: shortest distance between posterior ocellus and eye = 1.0: 1.8: 1.8. Vertex and temple shiny and with sparse setosity, with mid-longitudinal groove. Occipital carina weakly curved dorsally.

***Mesosoma.*** Mesosoma 2.0 × longer than high. Entire mesosoma coriaceous and setose. Mesopleuron and metapleuron setose. Mesoscutum shiny, without groove medially, sparsely punctate, notauli present. Scutellar sulcus smooth with strong medial carina and a pair of incomplete submedial carinae. Axillae narrow, finely striate. Raised anterodorsal area of mesopleuron below subalar depression with strong punctures tending to merge into distinct diagonal striate anteriorly. Precoxal sulcus complete, sinuate, transversely striate, broad and weakly impressed anteriorly becoming narrower and deeper to posterior margin of mesopleuron. Propodeum with submedial carinae anteriorly weakly curved and converging posteriorly; with strong setiferous punctures lateral to them, reticulate with long setae and without a mid-longitudinal carina.

***Wings.*** Fore wing. Lengths of fore wing veins r-rs: 3RSa: 3RSb = 1.0: 2.7: 3.7. Lengths of vein 2RS: 3RSa: rs-m = 1.7: 2.0: 1.0.

***Legs.*** Lengths of fore femur: fore tibia: fore tarsus = 1.1: 1.0: 1.1. Lengths of hind femur: hind tibia: hind tarsus = 1.0: 1.1: 1.4. Length of hind femur and tibia 2.7 × and 6.8 × as long as wide, respectively. Hind tibial spurs with long setae on basal 0.3. Tarsal claws with large acutely pointed basal lobe.

***Metasoma.*** T1 1.5 × longer than posteriorly wide. T2 1.5 × as long as T3. TT1 and 2 with mid-longitudinal carina dorsally, sparsely striate. TT3–6 with distinct striate sculpture. Ovipositor sheath straight and much shorter than hind basitarsus, ~ 0.2 × length hind femur (including trochantellus).

***Colour.*** Bicoloured body; scapus, pedicellus and flagellar segments dark brown. Head brownish black, but mouthparts area ivory, stemmaticum dark brown. Mesosoma dark brown to black except for ivory tegulae. Metasoma bicoloured; T1 and T6 ivory, T2 anteriorly and laterally ivory with black mark in the middle, TT3–5 mainly black, hypopygium white. Wings hyaline with dark brown venation, pterostigma brown. Fore legs and mid legs bicoloured brownish and white, hind legs entirely black. Ovipositor and ovipositor sheath black.

**Male.** Length of body 5.9 mm, of fore wing 4.9. Antenna incomplete, with at least 38 flagellomeres. Sculpture on frons less strong. Occipital carina distinctly pointed medially. Head yellow, darker dorsally. Mesosoma orange-yellow except mesopleuron, mesosternum, metapleuron and propodeum which are largely brown to piceous. Metasoma cream-white except T3 behind anterior transverse groove with pair or large black marks the join narrowly across midline and project to posterolateral margin, and T4 which same pattern as T3 except black marks not connected medially.

##### Variation (female).

Antennae with 49–52 flagellomeres.

##### Distribution.

Northern Thailand and Eastern Thailand.

##### Hosts.

Unknown.

##### Etymology.

This species is named after the third author’s husband.

#### ﻿ Troporhogas hugoolseni

Taxon classificationAnimaliaHymenopteraBraconidae

Quicke, Loncle & Butcher sp. nov.

370DAF8A-F10F-5263-8995-0B4EDC0AD759

https://zoobank.org/3A8D97AC-40B4-44E9-9C4E-DE3734416B3E

Figs 13 , 14 , 21B

##### Type material.

***Holotype*** ♀, Thailand, Nakhon Ratchasima province, Khoa Yai National Park, 19.iv.2007, 14°25.535'N, 101°23.442'E, Malaise trap, col. S. Warat (QSBG).

**Figure 13.**
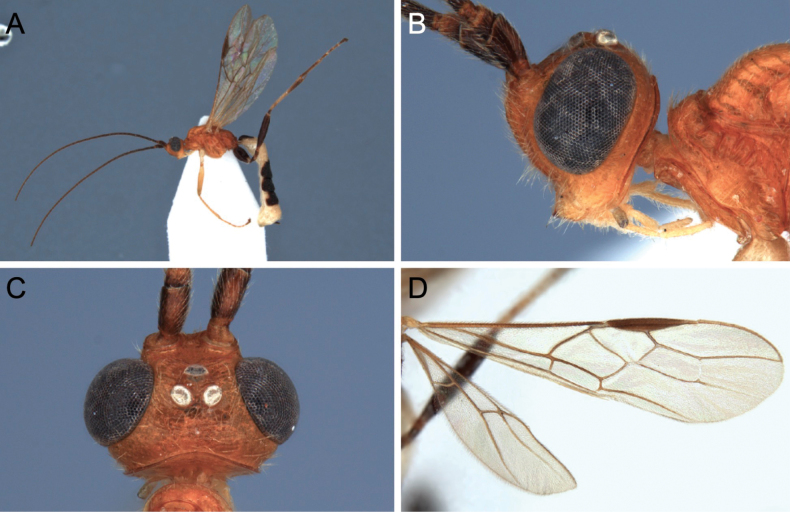
Light micrographs of holotype female *T.hugoolseni* sp. nov. **A** habitus, lateral view **B** head, lateral view **C** head, dorsal view **D** fore wing.

##### Diagnosis.

*Troporhogashugoolseni* sp. nov. can be differentiated from others with similar colour pattern (orangish mesosoma, T1 with black mark anteriorly, T2 largely and T6 entirely white) by having the vertex smooth except for small setiferous punctures and the raised oblique area of mesopleuron immediately below subalar depression moderately densely punctate but without oblique striation.

**Figure 14.**
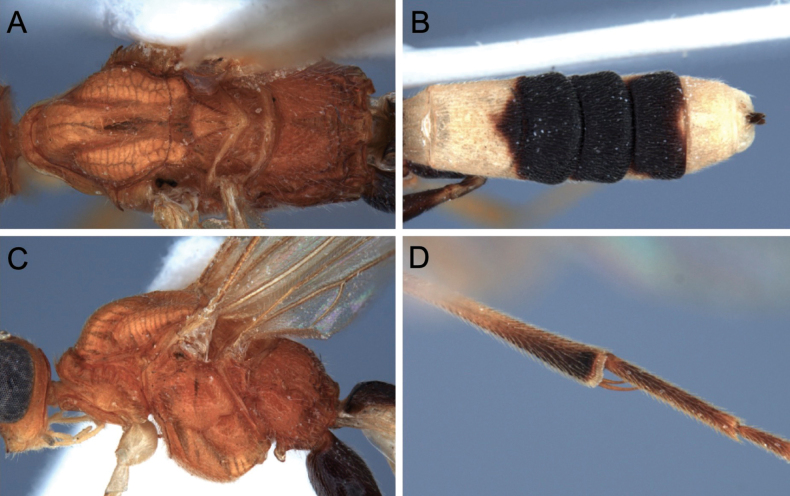
Light micrographs of holotype female *T.hugoolseni*, sp. nov., **A**, mesosoma, dorsal view **B** metasoma, dorsal view **C** mesosoma, lateral view **D** hind tibial spurs.

##### Description.

Holotype, female body length 6.2 mm; fore wing 5.5 mm; ovipositor sheath 0.5 mm.

***Head.*** Antenna incomplete, with at least 40 flagellomeres. First flagellomere 2.0 × longer than second and third, respectively. Width of head: width of face: height of eye = 3.1: 1.6: 1.0. Shortest distance between posterior ocelli: transverse diameter of posterior ocellus: shortest distance between posterior ocellus and eye = 1.0: 1.4: 1.6. Face and clypeus rugose with sparse setosity laterally. Vertex and temple shiny and with sparse setosity. Occipital carina complete, curved dorsally.

***Mesosoma.*** Mesosoma 1.6 × longer than high. Mesoscutum shiny, sparsely punctate, with narrow foveate longitudinal groove mid-posteriorly. Scutellar sulcus with three carinae between outer pair. Axillae striate. Raised anterodorsal area of mesopleuron below subalar depression with diagonal striation. Precoxal sulcus short, broad, and shallow, with weak irregular striation. Mid-longitudinal carina of metanotum strong and roundly protruding posteriorly, weak anteriorly. Propodeum reticulate with long setae, with submedial carinae meeting medially in V-shape connected by ladder of variably developed transverse crenulations, lateral to these with moderately dense, small setiferous punctures.

***Wings.*** Fore wing. Lengths of fore wing veins r-rs: 3RSa: 3RSb = 1.0: 2.8: 4.7. Lengths of vein 2RS: 3RSa: rs-m = 1.1: 1.9: 1.0.

***Legs.*** Lengths of fore femur: fore tibia: fore tarsus = 1.0: 1.1: 1.5. Lengths of hind femur: hind tibia: hind tarsus = 1.0: 1.3: 1.6 Length of hind femur and tibia 5.0 × and 8.3 × as long as wide, respectively. Hind tibial spurs glabrous. Tarsal claws with large acutely pointed basal lobe.

***Metasoma.*** T1 1.1 × longer than posteriorly wide. T2 1.5 × as long as T3. TT1 and 2 with mid-longitudinal carina dorsally, sparsely striate. TT3−6 with distinct striate sculpture and without medial longitudinal carina. Ovipositor sheath straight and shorter than hind basitarsus, ~ 0.3 × length hind femur (including trochantellus).

***Colour.*** Body tricoloured; scapus, pedicellus and flagellar segments dark brown. Head including stemmaticum orange-red, mouthparts paler brownish yellow. Mesosoma and tegulae ochraceous. Metasoma bicoloured; TT1, 2, and 6 white but T1 and T6 with small black marks anteriorly and posteriorly, TT3–5 black with small white anterolateral areas and with small white spot on the mid-posterior margin of T5, hypopygium white. Wings hyaline with dark brown venation, pterostigma brown. Fore legs and mid legs yellow, hind legs mainly dark brown. Ovipositor and ovipositor sheath black.

**Male.** Unknown.

##### Distribution.

Central Thailand.

##### Hosts.

Unknown.

##### Etymology.

This species named after Mr Hugo Olsen, Norwegian marathoner and a special friend of the corresponding author.

#### ﻿ Troporhogas rafaelnadali

Taxon classificationAnimaliaHymenopteraBraconidae

Quicke, Loncle & Butcher sp. nov.

0AF5DA0F-9941-5846-B820-C266D23C6052

https://zoobank.org/58BBFD86-4131-40ED-8D99-C900717A8B48

Figs 15 , 21G

##### Type material.

***Holotype*** ♀, Thailand, Nakhon Ratchasima province, Khao Yai National Park, 22.xii.2002, 19°14.459'N, 101°04.373'E, Malaise trap, col. S. Pong (QSBG).

**Figure 15.**
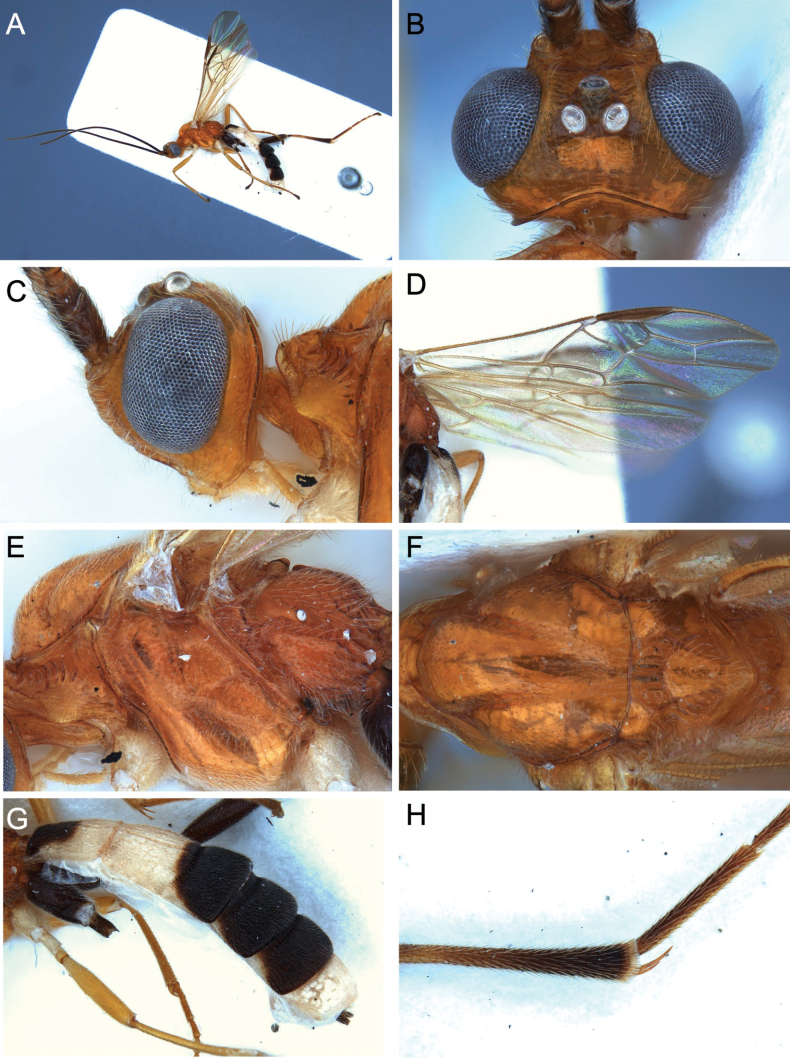
Light micrographs of holotype female *T.rafaelnadali* sp. nov., **A**, habitus, lateral view **B** head, dorsal view **C** head, lateral view **D** wings **E** mesosoma, lateral view **F** mesosoma, dorsal view **G** metasoma, oblique dorsal view **H** hind tibial spurs.

##### Diagnosis.

*Troporhogasrafaelnadali* sp. nov. can be distinguished from other members of the genus with similar colour pattern (orangish mesosoma, T1 with black mark anteriorly, T2 largely and T6 entirely white) by having the vertex transversely striate and the raised oblique area of mesopleuron immediately below subalar depression strongly finely striate.

##### Description.

Holotype, female, body length 6.0 mm; fore wing 4.2 mm; ovipositor sheath 0.5 mm.

***Head.*** Antenna with 48 flagellomeres. First flagellomere 1.6 × longer than second and third, respectively. Width of head: width of face: height of eye = 3.6: 1.8: 1.0. Shortest distance between posterior ocelli: transverse diameter of posterior ocellus: shortest distance between posterior ocellus and eye = 1.0: 2.3: 1.3. Face and clypeus rugose with sparse setosity laterally. Vertex and temple shiny and smooth. Occipital carina completely present, slightly pointed dorsally.

***Mesosoma.*** Mesosoma 1.7 × longer than high. Antescutal depression relatively well developed. Mesoscutum smooth and shiny, sparsely punctate, with narrow, foveate groove mid-posteriorly. Scutellar sulcus smooth with three carinae between outer margins. Mesopleuron and metapleuron setose. Precoxal sulcus shallow, long narrow and down-curving anteriorly, with narrow band of crenulation. Median area of metanotum with strong, roundly protruding mid-longitudinal carina over median area, but carina weak anteriorly. Propodeum with submedial carinae anteriorly forming a marrow ‘V’-shape and at extreme anterior, a short mid-longitudinal carina, lateral to this with dense small setiferous punctation.

***Wings.*** Fore wing. Lengths of fore wing veins r-rs: 3RSa: 3RSb = 1.0: 1.2: 2.0. Lengths of vein 2RS: 3RSa: rs-m = 1.7: 2.0: 1.0.

***Legs.*** Lengths of fore femur: fore tibia: fore tarsus = 1.0: 1.1: 1.4. Lengths of hind femur: hind tibia: hind tarsus = 1.0: 1.1: 1.9. Length of hind femur and tibia 3.6 × and 6.3 × as long as wide, respectively. Hind tibial spurs glabrous. Tarsal claws with large acutely pointed basal lobe.

***Metasoma.*** T1 1.1 × longer than posteriorly wide. T2 1.3 × longer than third tergite, 0.5 × as long as T3. TT1 and 2 with mid-longitudinal carina dorsally, sparsely striate. TT1–3 with distinct striate sculpture and without medial longitudinal carina. Ovipositor sheath straight and shorter than hind basitarsus, ~ 0.1 × length hind femur (including trochantellus).

***Colour.*** Body tricoloured body orange-yellow, black, and white. Scapus, pedicellus, and flagellar segments brown. Head and mouthparts, including palps, orange-yellow. Mesosoma completely orange-yellow except for whitish tegula. Metasoma tricoloured: TT1 and 2 white with small black mark anteriorly, TT3–5 black except for small white anterolateral areas, T6 white. Wings hyaline with dark brown venation distally becoming paler basally, pterostigma dark brown. Fore and mid legs brownish yellow with coxa and trochanter white, hind leg black except basal 0.6 of tibia brown.

**Male.** Unknown.

##### Distribution.

Central Thailand.

##### Biology.

Unknown.

##### Etymology.

This species is named after a Spanish professional tennis player, Rafael Nadal who has been ranked world No. 1 in singles by the ATP (Association of Tennis Professionals).

#### ﻿ Troporhogas rogerfedereri

Taxon classificationAnimaliaHymenopteraBraconidae

Quicke, Loncle & Butcher sp. nov.

A256A384-C6CD-5A74-AF15-03F29709BBC4

https://zoobank.org/04E5147A-1A14-463F-9C6C-8B2BC83D7A54

Figs 16 , 21E

##### Type material.

***Holotype*** ♀, Thailand, Surat Thani province, Khao Sok NP, Klong Morg unit, 23.vi.2009, 84m, Malaise trap, coll. Pongphan (QSBG).

##### Diagnosis.

**Figure 16.**
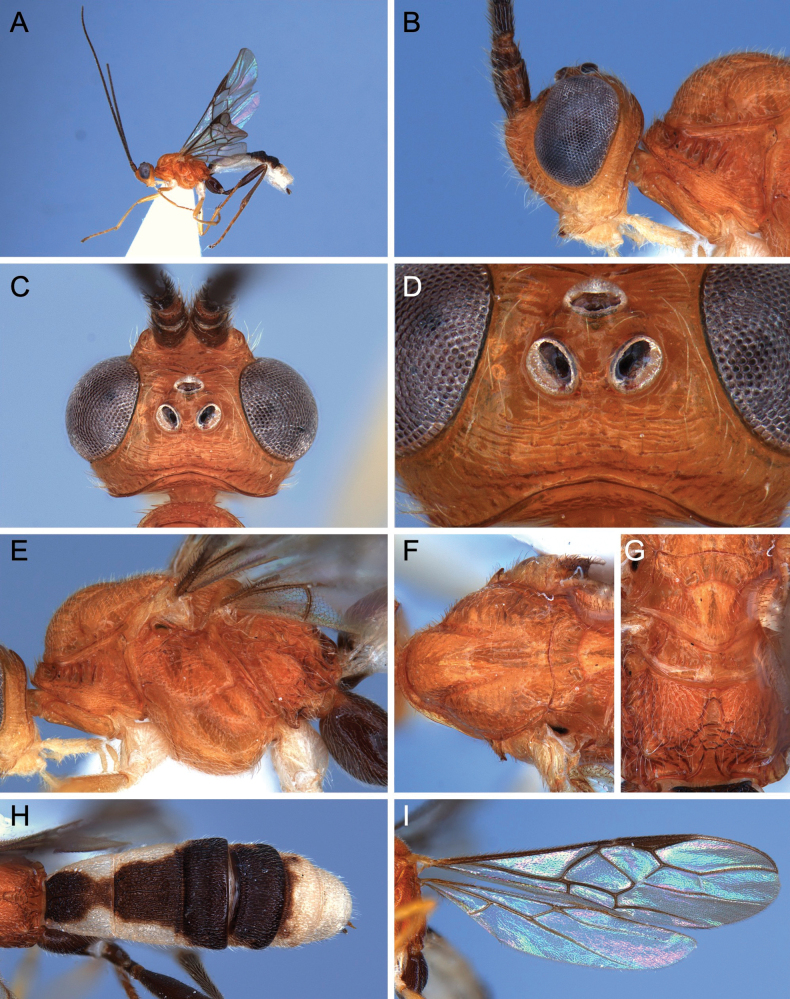
Light micrographs of holotype female *T.rogerfedereri* sp. nov. **A** habitus, lateral view **B** head and anterior mesosoma, lateral view **C** head, dorsal view **D** stemmaticum and vertex, detail **E** mesosoma, lateral view **F** anterior mesosoma, dorsal view **G** scutellum and propodeum, dorsal view **H** metasoma, dorsal view **I** wings.

*Troporhogasrogerfedereri* sp. nov. is the only Thai species know with the submedial propodeal carinae forming a wide U−shape anteriorly (Fig. 16G). It differs from the only other known species with this feature, *T.flavistigma*, in having the vertex strongly transversely striate.

##### Description.

Holotype, female, body length 6.0 mm; fore wing 4.2 mm; ovipositor sheath 0.5 mm.

***Head.*** Antenna with 40 flagellomeres. Terminal flagellomere acuminate, 4.5 × longer than wide. Penultimate flagellomere 3.0 × longer than wide. First flagellomere 1.0 × and 1.05 × longer than the second and third, respectively, the latter 2.4 × longer than wide. Width of face: width of head: height of eye = 1.0: 2.3: 1.4. Face transversely striate with a smooth longitudinal ridge medially. Inter-tentorial distance 2.3 × tentorio-ocular distance. Carina running from anterior margins of antennal sockets well-developed and uniting medially anterior of median ocellus. Shortest distance between posterior ocelli: transverse diameter of posterior ocellus: shortest distance between posterior ocellus and eye = 1.0: 1.4: 1.0. Vertex and temple transversely striate. Occipital carina strong, more or less evenly and weakly curved, with deep transverse groove immediately in front of it.

***Mesosoma.*** Mesosoma 1.5 × longer than high. Antescutal depression narrow. Mesopleuron and metapleuron setose. Mesoscutum shiny, without groove posteromedially, sparsely punctate. Scutellar sulcus with five carinae between outer pair. Raised anterodorsal area of mesopleuron below subalar depression with diagonal striation. Precoxal sulcus shallow, short, upcurved posteriorly, with weak irregular sculpture. Propodeum with pair of submedial carinae forming a wide inverted ‘U’-shape anteriorly; lateral to the carinae anteriorly with deep punctures that form close longitudinal rows, posteriorly with coarse irregular reticulations.

***Wings.*** Fore wing. Lengths of fore wing veins r-rs: 3RSa: 3RSb = 1.0: 3.0: 5.3. Lengths of vein 2RS: 3RSa: rs-m = 1.2: 2.3: 1.0.

***Legs.*** Lengths of fore femur: fore tibia: fore tarsus = 1.0: 1.1: 1.2. Lengths of hind femur: hind tibia: hind tarsus = 1.0: 1.1: 1.3. Length of hind femur and tibia 5.8 × and 15.0 × as long as medially wide, respectively. Hind tibial spurs distinctly setose on basal third. Tarsal claws with large acutely pointed basal lobe.

***Metasoma.*** T1 as long as posteriorly wide, T2 1.4 × longer than T3. TT1 and 2 sparsely striate, with mid-longitudinal carina. TT3–6 with distinct striate sculpture. Ovipositor sheath straight and shorter than hind basitarsus, ~ 0.3 × length of hind femur (including trochantellus).

***Colour.*** Body tricoloured; scapus, pedicellus and flagellar segments dark brown to black. Head orange-red, and mouthparts paler ochraceous, stemmaticum ochraceous. Mesosoma orange-red, tegulae paler ochraceous. Front and middle legs brownish yellow, except coxae and trochanters white, fore tarsus somewhat darker; hind leg largely black, tarsus paler. Wings hyaline with dark brown venation that becomes distinctly more yellowish towards wing base, pterostigma dark brown. Metasoma bicoloured; TT1–4 white with contiguous medial piceous to black marks that are narrowest at the junction of TT1 and 2; T5 largely except anteriorly, and T6 entirely ivory white; hypopygium white.

**Male.** Unknown.

##### Distribution.

Peninsular Thailand.

##### Biology.

Unknown.

##### Etymology.

This species is named after a Swiss former professional tennis player, Dr. Roger Federer who was ranked world No.1 in singles by the ATP.

### ﻿New record of *Troporhogas* from Thailand

Two new species records for Thailand were identified by key to species of the genus from Vietnam (Long 2014) and then, detailed comparison the specimens with original photographs of the type specimens from Vietnam.

#### ﻿ Troporhogas contrastus

Taxon classificationAnimaliaHymenopteraBraconidae

(Long, 2014)

A9D7DA50-69DA-5F02-A987-99428A30F479

Figs 17 , 21I

##### Material.

Thailand, **Nakorn Ratchasima province**, 6♀ Sakaerat Environmental Research Station (SERS); Light trap; 10–13.v.2021, 17 iii 2021 (CUMZ); **Nakorn Ratchasima province**, 3♂ Sakaerat Environmental Research Station (SERS), light trap; 17.ii–20.iii.2021 (CUMZ); **Chiang Mai province**, 1♂ Doi Chiangdao, 9–16.xi.2007, Malaise trap, (CUMZ); 1♀; Doi Phahompok, 11–18 vii 2007, Malaise trap (CUMZ); **Uthai Thani province**, 1♀; Khao Yong Rum, 6–8.vi.1986, 400 m, coll. J.M. Allen (NHMUK).

**Figure 17.**
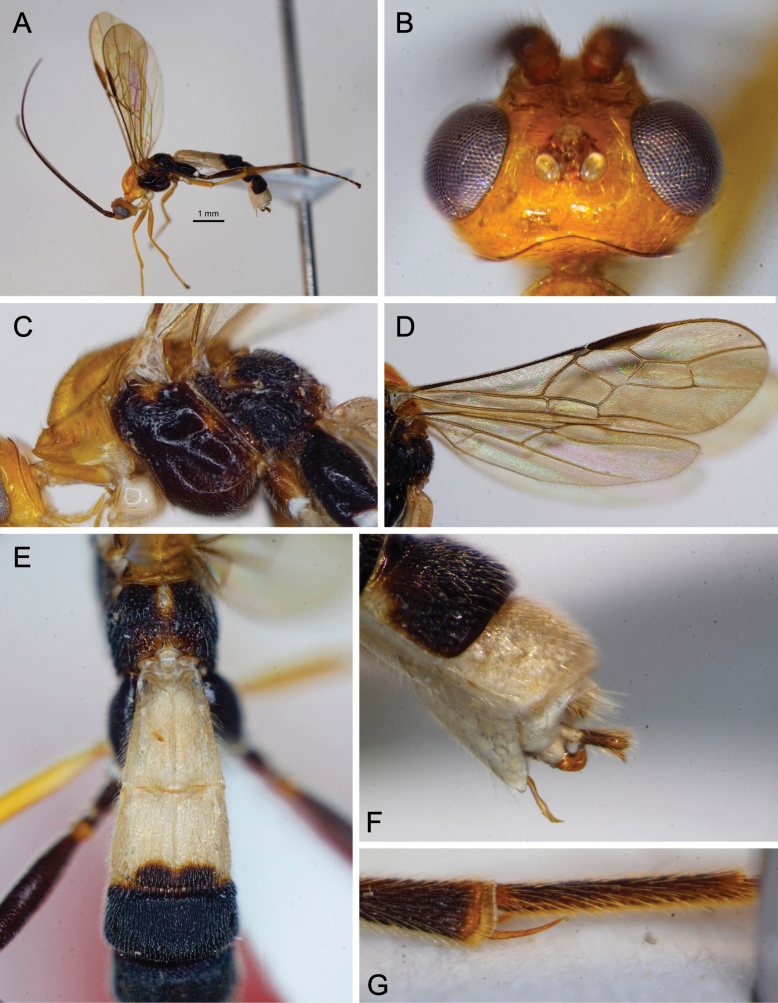
Light micrographs *T.contrastus* holotype female from Vietnam **A** habitus, lateral view **B** head, dorsal view **C** mesosoma, lateral view **D**, wings E propodeum and anterior metasoma, dorsal view **F** TT5 and 6, lateral view **G** hind tibial spurs.

##### Diagnosis.

**Female.** Body length 6.4–7.4 mm, length of fore wing 5.4–5.5 mm, antenna 7.9 mm; head in dorsal view occipital carina weakly curved, compound eyes large, lateral view temple and malar space small, face with median carina, frons smooth with sparse setae, maxillary palp 5-segmented; mesoscutum shiny and smooth with fine setae, notauli deep, propodeum areolate medially, rugose anteriorly and reticulated posteriorly; fore wing: pterostigma pale brown, vein r-rs arising slightly before the middle of pterostigma, (RS+M)a straight, hind wing: m-cu absent, cu-a reclivous; length of inner hind tibial spur < 1/3 of basitarsus, hind tarsal claw with large lobe; TT1 and 2 with dorsal longitudinal carina enclosing a triangular area at the base, metasomal tergites densely striate, ovipositor short; head yellow but stemmaticum black, mesosoma yellow except for meso- and metapleuron and propodeum black, legs yellow but hind legs brown, metasoma ivory, except for TT3–5 blackish brown, T2 with black spot posteriorly.

**Male.** Length of body 5.0–5.1 mm, of fore wing 4.9. Antenna with 36–39 flagellomeres. Sculpture on frons less strong. Occipital carina more or less rounded medially. Head and mesosoma orange-yellow except mesopleuron sometimes with dark medial mark, metapleuron largely piceous or black, propodeum black. Hind leg largely black but tarsus dark brown. Metasoma cream-white except TT3 and 4 which variably have a piceous to black mark submedially, to largely black behind anterior transverse groove with a weak break indicated anteriorly.

##### Variation.

One female specimen has the anterior third of middle lobe of mesoscutum piceous.

##### General distribution.

North central and north west Vietnam (Long 2014), north and central Thailand.

##### Biology.

Unknown.

#### ﻿ Troporhogas tricoloratus

Taxon classificationAnimaliaHymenopteraBraconidae

(Long, 2014)

2E2F68D6-7A39-5404-8B9B-219E16300D7B

Figs 18 , 21D

##### Material.

Thailand • 1♀, Phetchabun province, Nam Nao National Park, 16°13.103'N, 101°33.836'E, 28.iv.2007, Malaise trap, col. J. Leng (QSBG); 1♂, Sara Buri province, Kaeng Khoi, Chulalongkorn University Campus, 27.xii.2016, 15.589°N, 101.011°E, light trap, col. Mudang Marissa (CUMZ).

**Figure 18.**
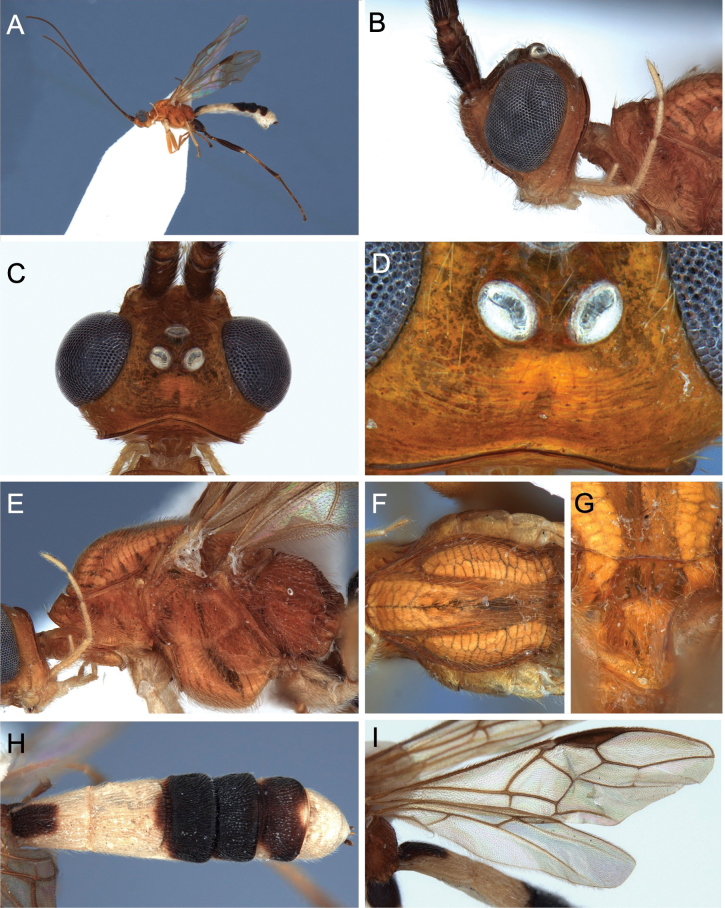
Light micrographs of female Thai specimen of *T.tricoloratus***A** habitus, lateral view B head, lateral view **C** head, dorsal view **D** vertex, dorsal view **E** mesosoma, lateral view **F** mesoscutum, dorsal view **G** scutellar sulcus **H** metasoma, dorsal view **I** wings.

##### Re-description.

**Female**, body length 7.1 mm; fore wing 5.0 mm; ovipositor sheath 0.6 mm.

***Head.*** Antenna with 45 flagellomeres. First flagellomere 1.4 × longer than second and third, respectively. Width of head: width of face: height of eye = 2.6: 1.2: 1.0. Shortest distance between posterior ocelli: transverse diameter of posterior ocellus: shortest distance between posterior ocellus and eye = 1.0: 1.8: 1.8. Face and clypeus rugose with sparse setosity laterally. Vertex and temple shiny and with striate sculptured. Occipital carina completely present, curved dorsally.

***Mesosoma.*** Mesosoma 1.5 × longer than high, smooth and shiny. Mesopleuron and metapleuron setose. Mesoscutum shiny, with narrow, punctate median groove posteriorly, sparsely punctate. Scutellar sulcus smooth without carina. Axillae striate. Raised anterodorsal area of mesopleuron below subalar depression smooth. Precoxal sulcus, short and crenulate. Propodeum with submedial carinae anteriorly forming very narrow, sharp V-shape, lateral to this with rather dense and strong setiferous punctation.

***Wings.*** Fore wing. Lengths of fore wing veins r-rs: 3RSa: 3RSb = 1.0: 2.2: 4.3. Lengths of vein 2RS: 3RSa: rs-m = 1.0: 2.2: 1.2.

***Legs.*** Lengths of fore femur: fore tibia: fore tarsus = 1.3: 1.2: 1.0. Lengths of hind femur: hind tibia: hind tarsus = 1.0: 1.3: 1.6. Length of hind femur and tibia 4.0 × and 7.5 × as long as wide, respectively. Hind tibial spurs glabrous. Tarsal claws with large acutely pointed basal lobe.

***Metasoma.*** T1 1.3 × longer than posteriorly wide. T2 1.4 × longer than tergite 3. TT1 and 2 with mid-longitudinal carina, sparsely striate. TT3–6 with distinct striate sculpture and without medial longitudinal carina. Ovipositor sheath straight and shorter than hind basitarsus, ~ 0.2 × length hind femur (including trochantellus).

***Colour.*** Body tricoloured: scapus, pedicellus and flagellar segments brown. Head and mouthparts area ochraceous, stemmaticum ochraceous. Mesosoma and tegulae ochraceous yellow colour. Metasoma bicoloured; TT1, 2, and 6 white but first tergite with small black marks anteriorly and sixth with very small black mark posteriorly, TT3–5 black (with a small anterolateral areas and spot on middle of posterior area margin of tergite 5 white), hypopygium white. Wings hyaline with dark brown venation, pterostigma brown. Fore legs and mid legs yellow, hind legs entirely black and dark brown. Ovipositor and ovipositor sheath black.

**Male.** Head and mesosoma orange-yellow. Metasoma ivory-white with black markings: TT1 and 2 with black marks meso-anteriorly, TT3 and 4 with large black marks reach anterior margin anteriorly and posterolateral corners posteriorly, but with large ivory antero-lateral areas, TT5 and 6 ivory-white. Wings hyaline with dark brown pterostigma and venation. Fore and mid legs orange-brown with coxa white and tarsus brown. Hind coxa black with large whitish dorsal patch, hind trochantellus white, hind femur white on basal 0.4, distally black, hind tibia white on basal 0.75, distally black, hind tarsus dark brown.

##### Distribution.

Northern Vietnam and northern Thailand and Eastern Thailand.

##### Hosts.

Unknown.

##### Notes.

The female Thai specimen agrees very well with the original description except that the dark mark at posterior of T2 is smaller and there is a small pale spot on the mid posterior margin of T2. Thus, given that there is some colour variation in nearly all the species represented by multiple individuals of the same sex, we believe them to be conspecific.

### ﻿Notes on *Troporhogas* from China

#### ﻿ Troporhogas chinensis

Taxon classificationAnimaliaHymenopteraBraconidae

(Chen & He, 1997)

B32203C8-60AF-533D-A3F7-F4E0271AC6C4

Figs 19A, B , 21B

##### Comments.

Known only from the holotype female from the southerly island of Hainan. It differs from *T.albilateralis* which also has contiguous black markings on TT1–5 in that the whitish anterolateral areas of the tergites are distinctly smaller (Fig. 21B cf. 21A), and the fore wing vein 1cu-a is virtually interstitial.

**Figure 19.**
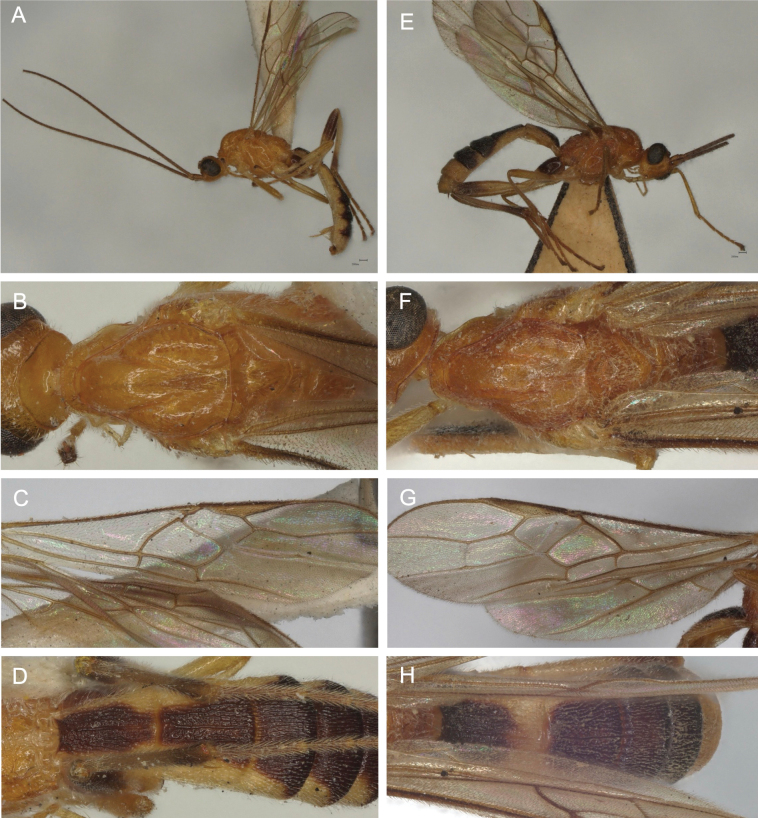
Light micrographs of Chinese species of *Troporhogas*. **A–D***T.chinensis*, ♀ holotype **A** habitus, lateral view **B** posterior of head and mesosoma, dorsal view **C** fore wing **D** metasoma, dorsal view **E–H***T.flavistigma* ♀ holotype **E** habitus, lateral view **F** posterior of head and mesosoma, dorsal view **G** fore wing **H** metasoma, dorsal view.

#### ﻿ Troporhogas flavistigma

Taxon classificationAnimaliaHymenopteraBraconidae

(Chen & He, 1997)

6335CAEC-83D1-50DD-AF5B-F436950753A4

Figs 19D–F , 21F

##### Comments.

Known from the holotype female, two female paratypes, and a male paratype collectively from locations in Fujian, Guangxi, and Yunnan. The original description notes some variation in colour with TT1 and 2 sometimes being largely yellow and T5 sometimes largely black but does not specify whether or not these variants reflect sexual dimorphism.

#### ﻿ Troporhogas guangxiensis

Taxon classificationAnimaliaHymenopteraBraconidae

(Chen & He, 1997)

A946B95B-CA9C-5E41-BFAC-D7B1C0971752

Fig. 20E–H

##### Comments.

This is one of two predominantly brown-yellow species that have a simple rounded, not produced, basal lobe to the claws (Chen and He 1997: fig. 324). The other known species with this unusual character is *T.simulatus*, thus far known only from Vietnam (Long 2014: fig. 31).

**Figure 20.**
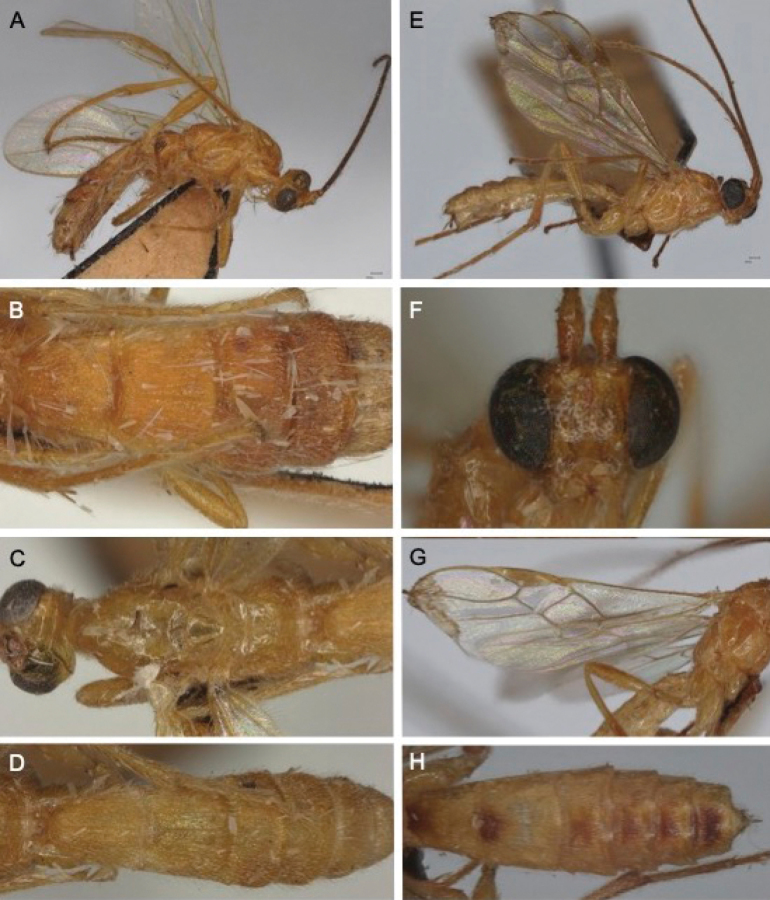
Light micrographs type specimens of Chinese species of *Troporhogas***A, B***T.rugiventris***A** habitus, lateral view **B** metasoma, dorsal view **C, D***T.unicolor***C** head and mesosoma, dorsal view **D** metasoma, dorsal view **E–H***T.guangxiensis***E** habitus, lateral view **F** head, anterior view **G** fore wing **H** metasoma, dorsal view.

**Figure 21.**
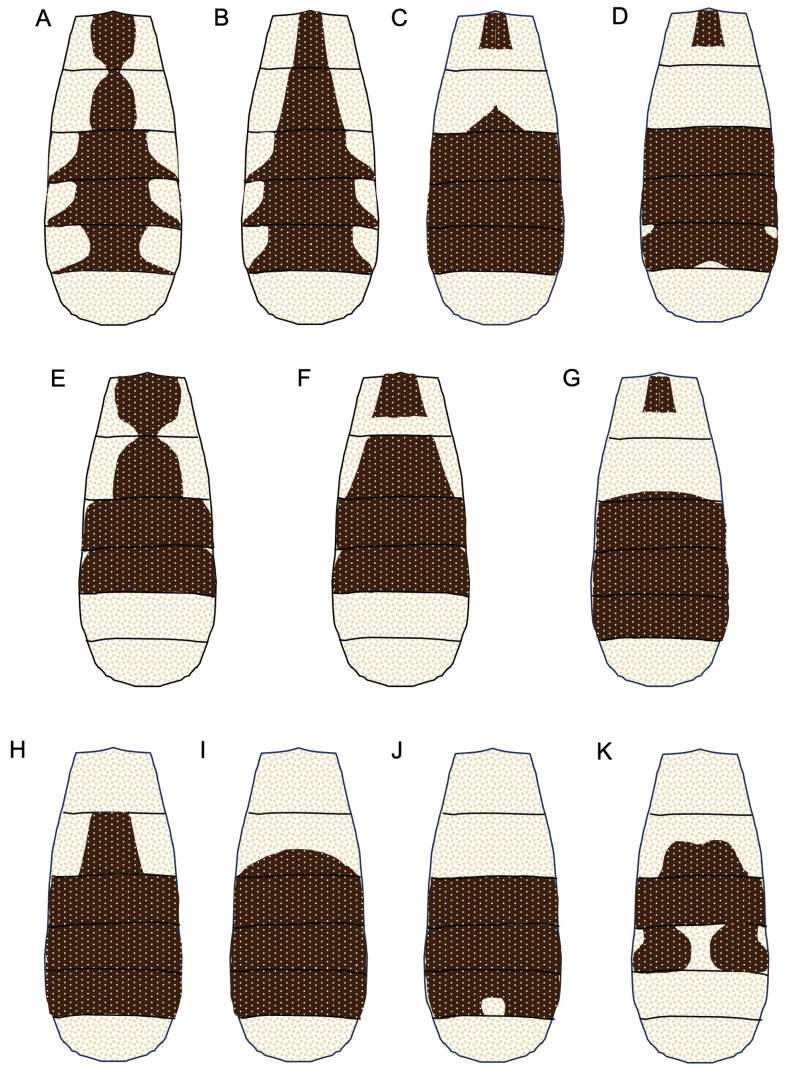
Drawings of metasomal colour patterns of females of *Troporhogas* species **A***T.albilateralis***B***T.chinensis***C***T.hugoolseni* sp. nov. **D***T.tricoloratus* (Thai specimen) **E***T.rogerfedereri* sp. nov. **F***T.flavistigma***G***T.rafaelnadali* sp. nov. **H***T.benjamini* sp. nov. **I***T.contrastus***J***T.alboniger* sp. nov. **K***T.anamikae* sp. nov.

#### ﻿ Troporhogas rugivertex

Taxon classificationAnimaliaHymenopteraBraconidae

(Chen & He, 1997)

AA40623C-F7A8-5019-A014-4C54F0F0DF5E

Fig. 20A, B

##### Comments.

Known only from holotype female from Yunnan. It is the only known entirely ochreous yellow species with uniformly hyaline wings and a dorsomedially pointed occipital carina.

#### ﻿ Troporhogas unicolor

Taxon classificationAnimaliaHymenopteraBraconidae

(Chen & He, 1997)

AA8EF6F8-59FA-5141-B1DE-5D89DE4E6CCC

Fig. 20C, D

##### Comments.

Known only from a single male specimen from Yunnan. The holotype has a distinctively coloured metasoma, ochreous yellow with some darker medial spots but, given the sexual dimorphism in colour pattern in the boldly coloured species (*T.benjamini* sp. nov. and *T.contrastus*), it is impossible to know whether this would be useful for recognising females of the species.

## ﻿Discussion

The molecular phylogenetic analysis (Fig. 4) shows that *Troporhogasalboniger* sp. nov. is nested within the *T.contrastus* clade, their barcode sequences differing by only one base pair. Hence all specimens of these two species fall into the same BIN (Ratnasingham and Hebert 2013), and they are undoubtedly closely related and presumably have speciated quite recently. Unfortunately for the new species we were only able to obtain sequences from the barcode gene and not from the other three markers. There are three reasons we recognise these as different species. Firstly, we identified three consistent differences in mesosomal sculpture, and importantly, the vertical striation in the new species at the anterior of the mesopleuron is not simply a matter of an increase in density of a feature as there is no indication of such sculpture in *T.contrastus*. Secondly, the difference in colouration is also not simply a matter of intensity. The available material of *T.contrastus* displays a small amount of colour variation, with some having the mesoscutum more orange-red and others black with piceous orange, but none show the pure blackness of *T.alboniger* sp. nov. The head, including mouthparts and palps, of *T.contrastus* are orange-yellow whereas in the new species the head is bicoloured with a sharp demarcation between the pure black upper parts and the virtually pure white malar area, mouthparts, and palps. Thirdly, there is very little variation in colour pattern in either of the two species that are represented in our collection based on multiple female specimens *T.benjamini* sp. nov. and *T.contrastus*.

Although a fairly gradual variation (especially in degree of melanisation) is not uncommon in the Braconidae, and examples are known of temperature, latitudinal (Ito et al. 2015), or host-induced colour differences, again in degree of melanisation: there are very few examples where genuine pattern polymorphism has been found (Tucker and Sharkey 2016). We are not aware of any examples involving the replacement of one colour with two quite different colours such as that seen in the difference between *T.contrastus* and *T.alboniger* sp. nov., and therefore we believe that collectively the evidence supports that these are two distinct species.

## Supplementary Material

XML Treatment for Troporhogas

XML Treatment for Troporhogas alboniger

XML Treatment for Troporhogas anamikae

XML Treatment for Troporhogas benjamini

XML Treatment for Troporhogas hugoolseni

XML Treatment for Troporhogas rafaelnadali

XML Treatment for Troporhogas rogerfedereri

XML Treatment for Troporhogas contrastus

XML Treatment for Troporhogas tricoloratus

XML Treatment for Troporhogas chinensis

XML Treatment for Troporhogas flavistigma

XML Treatment for Troporhogas guangxiensis

XML Treatment for Troporhogas rugivertex

XML Treatment for Troporhogas unicolor

## ﻿Additional information

### Author ORCIDs

Donald L. J. Quicke https://orcid.org/0000-0003-4471-6775

A. P. Ranjith https://orcid.org/0000-0001-7061-9659

Marisa K. Loncle https://orcid.org/0000-0002-6221-7331

Cornelis Van Achterberg https://orcid.org/0000-0002-6495-4853

Khuat Dang Long https://orcid.org/0000-0002-9237-7344

Buntika A. Butcher https://orcid.org/0000-0002-0541-0709

### Data availability

All of the data that support the findings of this study are available in the main text or Supplementary Information.

## Supplementary materials

Supplementary material 1 Provenances of sequenced specimens, DNA barcode index numbers (BINs), and GenBank accessions number Data type docx https://binary.pensoft.net/file/1085673This dataset is made available under the Open Database License (http://opendatacommons.org/licenses/odbl/1.0/). The Open Database License (ODbL) is a license agreement intended to allow users to freely share, modify, and use this Dataset while maintaining this same freedom for others, provided that the original source and author(s) are credited.Donald L. J. Quicke, A. P. Ranjith, Marisa K. Loncle, Cornelis Van Achterberg, Khuat Dang Long, Buntika A. Butcher
