# Supplementary material for: ﻿Revision of Troporhogas Cameron (Hymenoptera, Braconidae, Rogadinae) with six new species from India and Thailand
Source: Zookeys. 2024 Jul 5;1206:99–136. doi: 10.3897/zookeys.1206.120824 (PMC11245641; doi:10.3897/zookeys.1206.120824)
Supplement: Supplementary material 1 — Provenances of sequenced specimens, DNA barcode index numbers (BINs), and GenBank accessions number [file zookeys-1206-099_article-120824__-s001.docx]

| Genus | Species | Voucher_code | Process_ID | BOLD:BIN | Provenance | Genbank  CO1 | Genbank  Cytb | Genbank  16S | Genbank  28S |
| --- | --- | --- | --- | --- | --- | --- | --- | --- | --- |
| *Troporhogas* | *alboniger* n.sp. | CCDB-27842-E3 | BBTH1810-19 | AAH8865 | Thailand | PP301989 | - | - | - |
| *Troporhogas* | *alboniger* n.sp | BCLDQ1075 | ASQSQ372-09 | AAH8865 | Thailand | PP301990 | - | - | - |
| *Troporhogas* | *anamikae* n.sp. | CCDB-06326-C12 | BBTH2693-21 | AEO3062 | India | PP316977 | PP665746 | PP356625 | PP356641 |
| *Troporhogas* | *benjamini* n.sp. | CCDB-24264-B11 | BBTH2775-21 | - | Thailand | PP302000 | PP665752 | - | PP356654 |
| *Troporhogas* | *benjamini* n.sp. | CCDB-24264-A11 | BBTH2787-21 | - | Thailand | PP302001 | PP665749 | PP356635 | PP356651 |
| *Troporhogas* | *benjamini* n.sp. | CCDB-24264-A12 | BBTH2764-21 | - | Thailand | PP302002 | PP665750 | PP356636 | PP356650 |
| *Troporhogas* | *benjamini* n.sp. | BCLDQ01658 | ASQSR222-11 | AAL7148 | Thailand | JN278383 | - | PP356637 | - |
| *Troporhogas* | *benjamini* n.sp. | CCDB-24264-C12 | BBTH2788-21 | - | Thailand | PP302003 | PP665760 | PP356638 | PP356653 |
| *Troporhogas* | *contrastus* | CCDB-24264-B12 | BBTH2776-21 | - | Thailand | PP301991 | PP665753 | PP356626 | PP356642 |
| *Troporhogas* | *contrastus* | CCDB-24264-C07 | BBTH2783-21 | - | Thailand | PP301993 | PP665756 | PP356629 | PP356644 |
| *Troporhogas* | *contrastus* | CCDB-24264-C05 | BBTH2781-21 | - | Thailand | PP301992 | PP665754 | PP356627 | PP356643 |
| *Troporhogas* | *contrastus* | CCDB-24264-C06 | BBTH2782-21 | - | Thailand | PP301994 | PP665755 | PP356628 | PP356645 |
| *Troporhogas* | *contrastus* | CCDB-24264-C11 | BBTH2787-21 | - | Thailand | PP301995 | PP665759 | PP356632 | - |
| *Troporhogas* | *contrastus* | CCDB-24264-C08 | BBTH2784-21 | - | Thailand | PP301996 | PP665757 | PP356630 | PP356646 |
| *Troporhogas* | *contrastus* | CCDB-24264-A10 | BBTH2762-21 | - | Thailand | PP301998 | PP665748 | PP356633 | PP356649 |
| *Troporhogas* | *contrastus* | CCDB-24264-B10 | BBTH2774-21 | - | Thailand | PP301997 | PP665751 | PP356634 | PP356648 |
| *Troporhogas* | *contrastus* | CCDB-24264-C09 | BBTH2785-21 | - | Thailand | PP301999 | PP665758 | PP356631 | PP356647 |
| *Troporhogas* | *contrastus* | BCLDQ01660 | ASQSR224-11 | AAH8865 | Thailand | JN278385 | - | - | - |
| *Troporhogas* | *hugoolseni* n.sp. | BCLDQ01314 | ASQSP960-10 | AAL7148 | Thailand | HQ551251 | - | - | MT943591 |
| *Troporhogas* | *rafaelnadali* n.sp. | CCDB-24264-A06 | BBTH2758-21 | - | Thailand | PP302004 | PP665747 | PP356639 | PP356652 |
| *Troporhogas* | *rafaelnadali* n.sp. | BCLDQ01659 | ASQSR223-11 | AAU5966 | Thailand | JN278384 | - | - | - |
| *Troporhogas* | *rogerfedereri* n.sp. | BCLDQ01596 | ASQSR160-11 | AAU5967 | Thailand | JN278334 | - | - | - |
| Troporhogas | *ruficeps* | BCLDQ0785 | ASQSQ066-09 | - | Sri Lanka | MT639598 | - | - | - |
| *Troporhogas* | *tricoloratus* | CCDB-27844-F9 | BBTH783-17 | ADH6971 | Thailand | MT639377 | - | - | MW258965 |
| *Troporhogas* | *tricoloratus* | BCLDQ0769 | ASQSQ050-09 | ADH6971 | Thailand | MW259146 | - | - | PP356655 |
| *Troporhogas* | sp.1 | USNMENT00707640 | ASPNF145-12 | ACC8635 | Papua New Guinea | MT639526 | - | - | - |
| *Troporhogas* | sp.2 | BCLDQ01684 | ASQSR279-11 | ABA3874 | Congo | MT639583 | - | - | - |
| *Troporhogas* | sp.3 | JM863 | ASQBR534-09 | AAG5354 | Cameroon | JF963819 | - | - | - |
| *Troporhogas* | sp.4 | BCLDQ01686b | ASQBR992-20 | AEE8610 | Congo | MT639483 | - | - | - |
| *Troporhogas* | sp.5 | CCDB-27844-E12 | BBTH774-17 | ADH6640 | Madagascar | MT639372 | - | - | - |
| *Troporhogas* | sp.6 | USNMENT00681531 | ASQSP022-08 | AAG4997 | Papua New Guinea | HM373907 | - | - | - |
| *Rogasodes* | sp.1 | BCLDQ00773 | ASQSQ054-09 | AAH8799 | Thailand | JF962605 | - | - | JF903048 |
| *Rogasodes* | sp.2 | BCLDQ01167 | ASQSQ563-09 | AAH8995 | Thailand | HM435247 | - | MT606554 | EU854398 |
| *Rhogasella* | sp. | BCLDQ01633 | ASQSR197-11 | AAV2313 | Thailand | JN278361 | - | - | - |
|  |  |  |  |  |  |  |  |  |  |
